# Molecular Structure, Spectral Investigations, Hydrogen Bonding Interactions and Reactivity-Property Relationship of Caffeine-Citric Acid Cocrystal by Experimental and DFT Approach

**DOI:** 10.3389/fchem.2021.708538

**Published:** 2021-07-26

**Authors:** Priya Verma, Anubha Srivastava, Karnica Srivastava, Poonam Tandon, Manishkumar R. Shimpi

**Affiliations:** ^1^ Department of Physics, University of Lucknow, Lucknow, India; ^2^ Department of Physics, Isabella Thoburn (I.T.) College, Lucknow, India; ^3^ Department of Materials and Environmental Chemistry, Stockholm University, Stockholm, Sweden; ^4^ Chemistry of Interfaces, Luleå University of Technology, Luleå, Sweden

**Keywords:** caffeine-citric acid cocrystal, hydrogen bonding, structure, reactivity, property, characterization

## Abstract

The pharmaceutical cocrystal of caffeine-citric acid (CAF-CA, Form II) has been studied to explore the presence of hydrogen bonding interactions and structure-reactivity-property relationship between the two constituents CAF and Citric acid. The cocrystal was prepared by slurry crystallization. Powder X-ray diffraction (PXRD) analysis was done to characterize CAF-CA cocrystal. Also, differential scanning calorimetry (DSC) confirmed the existence of CAF-CA cocrystal. The vibrational spectroscopic (FT-IR and FT-Raman) signatures and quantum chemical approach have been used as a strategy to get insights into structural and spectral features of CAF-CA cocrystal. There was a good correlation among the experimental and theoretical results of dimer of cocrystal, as this model is capable of covering all nearest possible interactions present in the crystal structure of cocrystal. The spectroscopic results confirmed that (O33-H34) mode forms an intramolecular (C25 = O28∙∙∙H34-O33), while (O26-H27) (O39-H40) and (O43-H44) groups form intermolecular hydrogen bonding (O26-H27∙∙∙N24-C22, O39-H40∙∙∙O52 = C51 and O43-H44∙∙∙O86 = C83) in cocrystal due to red shifting and increment in bond length. The quantum theory of atoms in molecules (QTAIM) analysis revealed (O88-H89∙∙∙O41) as strongest intermolecular hydrogen bonding interaction with interaction energy −12.4247 kcal mol^−1^ in CAF-CA cocrystal. The natural bond orbital analysis of the second-order theory of the Fock matrix highlighted the presence of strong interactions (N∙∙∙H and O∙∙∙H) in cocrystal. The HOMO-LUMO energy gap value shows that the CAF-CA cocrystal is more reactive, less stable and softer than CAF active pharmaceutical ingredients. The electrophilic and nucleophilic reactivities of atomic sites involved in intermolecular hydrogen bond interactions in cocrystal have been demonstrated by mapping electron density isosurfaces over electrostatic potential i.e. plotting molecular electrostatic potential (MESP) map. The molar refractivity value of cocrystal lies within the set range by Lipinski and hence it may be used as orally active form. The results show that the physicochemical properties of CAF-CA cocrystal are enhanced in comparison to CAF (API).

## Introduction

Pharmaceutical cocrystals (Aakeröy and Sinha, 2018; Duggirala et al., 2016) have received interest in recent years from a variety of disciplines including chemical, material and pharmaceutical sciences. Cocrystals present an opportunity to enhance the physicochemical properties like solubility, stability, bioavailability, dissolution rates, compressibility and hygroscopicity without altering chemical structure of active pharmaceutical ingredients (API) (Corner et al., 2018; Alhalaweh et al., 2012; Jones et al., 2006; Shimpi et al., 2018; Bolla and Nangia, 2016). Generally, cocrystals rely on various intermolecular interactions like hydrogen bonding, π-π stacking and van der Waals forces. Among them hydrogen bonding interactions are crucial because of their energy parameters and directionality (Gamekkanda et al., 2018). The “best-donor-best-acceptor rule” governs the cocrystal formation by prioritizing the intermolecular hydrogen bonding interactions in the supramolecular (homo- and heteromeric) synthons (Etter, 1990). The supramolecular synthons may be strong (O-H∙∙∙O, N-H∙∙∙O, O-H∙∙∙N and N-H∙∙∙N) and weak (C-H∙∙∙O and C-H∙∙∙N) depending upon the geometries and arrangements of functional groups (Shattock et al., 2008; Vishweshwar et al., 2003).

**FIGURE 1:**
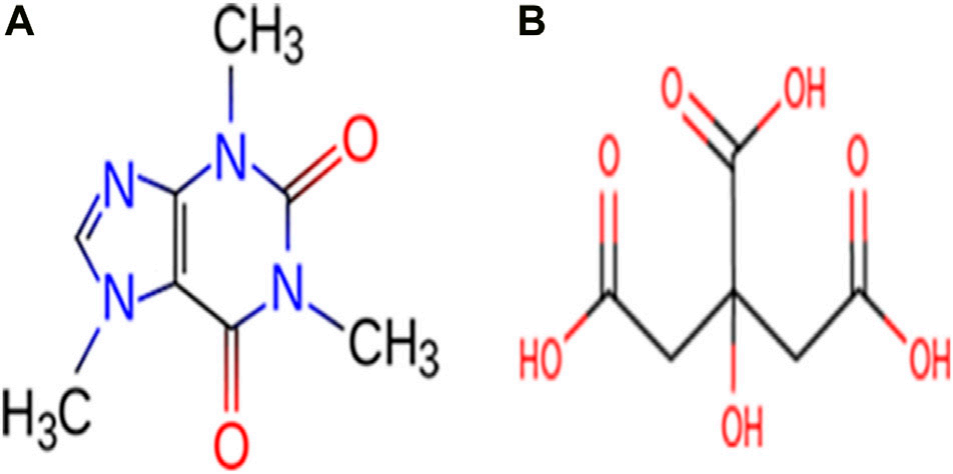
Molecular structures of **(A)** Caffeine and **(B)** Citric acid.

Caffeine (CAF) is slightly bitter, white crystalline, naturally occurring alkaloid. It belongs to the family of methylxanthine alkaloid and is a psychoactive stimulant drug which can be usually extracted from plants like coffee, guarana, cacao, yerba mate etc. (Pan et al., 2019). CAF can be used for the pharmaceutical drug preparations as an analgesic adjuvant, treat conditions like physical fatigue, drowsiness (Mou et al., 2019), apnea of prematurity (Philip et al., 2018) and bronchopulmonary dysplasia in premature infants (Principi et al., 2018). Citric acid (CA) is one of the most frequently used bioproduct which produces antiviral tissues and have buffering properties to control pH and production of antiviral tissues in pharmaceuticals (Ji et al., 2019; Chandramouli et al., 2016). It is capable of forming strong hydrogen bonding interactions with neighboring molecules, since it possesses both hydroxyl and carboxylic acid groups and can act as a hydrogen bond donor and acceptor. The molecular structures of CAF and CA are shown in Figure 1.

According to Leyssens et al. (Leyssens et al., 2014), cocrystals of CAF are useful to explore the fact that salt formation of CAF is not easily possible as it is a weak base. Cocrystals of caffeine with different coformers like oxalic, malonic, maleic, glutaric acids, methyl gallate have been studied by experimental and quantum chemical methods in order to improve physicochemical properties (Trask et al., 2006; Sun and Hou, 2008; Azizi et al., 2014). There are three CAF-CA cocrystal polymorphs reported in the literature (Smit and Hagen, 2015; Mukaida et al., 2015). In this work we investigated the most stable polymorph CAF-CA, Form II and denoted here as “CAF-CA” throughout the article. However, very limited studies (Smit and Hagen, 2015; Mukherjee et al., 2018) and no computational investigations using spectroscopic and DFT analysis are reported so far to explore the CAF-CA cocrystal.

In continuation to our previous work (Verma et al., 2019; Shukla et al., 2019), the present study includes the quantum chemical methods and spectroscopic techniques (Infrared, Raman spectra) to determine the vibrational spectroscopic signatures of monomer and dimer model of CAF-CA cocrystal. It is observed that dimer matches more closely to the CAF-CA cocrystal than monomer and isolated compounds due to the incorporation of nearest possible interactions. Additionally, powder X-ray diffraction (PXRD) and differential scanning calorimetry (DSC) techniques have been used to characterize the cocystal. The “Quantum theory of Atoms in molecules” (QTAIM) calculations (Kumar et al., 2016) provide the basis of understanding the conventional and non-conventional hydrogen bonding patterns in detail, which is indeed helpful to understand the behavior in CAF-CA cocrystal system. The natural bond orbital (NBO) analysis has been performed to study the several charge transfer interactions (lone pair; n → π*, n → σ* and σ → π*) and significant stabilization energies E^(2)^ within the CAF-CA cocrystalline system, which are important parameters to explain the stability of the molecule (Dunnington and Schmidt, 2012). The various properties of CAF, CA and CAF-CA cocrystal have been studied via electronic and chemical reactivity descriptors to predict the parameters like chemical reactivity, stability and site selectivity, which play crucial role in drug designing and its development (Elshakre et al., 2020). The molecular electrostatic potential surface (MESP) (Perera et al., 2016) has been plotted to study the electrophilic, nucleophilic reactivity and interactive behavior of CAF-CA cocrystal as well as of its two constituents CAF and CA. The Lipinski’s rule of five (Lipinski et al., 1997) has been applied to show the drug like behavior of CAF-CA cocrystal.

## Experimental Details

The two chemicals; CAF and CA were purchased from Sigma-Aldrich GmbH. A 10 ml-glass vial was charged with 1.9 g of CAF, 2.0 g of CA. The ethanol of 3 ml was added to form slurry. The reaction was allowed to stir for a total of 48 h, at which time the solids present were isolated by vacuum filtration and air dried at room temperature. The prepared CAF-CA cocrystal was analyzed by using the PXRD pattern and DSC analysis in order to validate the product material, as shown in Supplementary Figures S1, S2, respectively.

### Powder X-Ray Diffraction Pattern

PXRD patterns for the samples were collected using an Empyrean X-ray diffractometer (PANalytical, Netherlands) equipped with a PIXel3D detector and a monochromatic Cu K*α1* radiation X-Ray tube (*λ* = 1.54056 Å). The tube voltage and amperage were set at 45 kV and 40 mA, respectively. Samples were filled onto a metal sample holder and flattened. Instrument calibration was performed using a silicon reference standard. The sample was scanned 2θ range of 5–40°C, increasing at a step size of 0.026 at 25°C. The data were processed using high score plus software (PANalytical, Netherlands).

### Differential Scanning Calorimetry Analysis

DSC was performed using a PerkinElmer 6,000 instrument. The sample (about 2–4 mg) was placed into an aluminum DSC pan and the weight recorded accurately. The pan was covered with a lid and then crimped. The sample cell was heated under a nitrogen purge at a rate of 20 ml min^−1^ from −25°C to 200°C with heating speed of 10°C min^−1^. Indium metal was used as the calibration standard.

### Fourier Transform-Infrared Spectroscopy

The FT-IR spectrum of CAF-CA cocrystal was recorded on Bruker Vertex 80v FT-IR spectrometer equipped with a deuterated lanthanum α-alanine-doped triglycine sulfate (DLaTGS) detector and a platinum-attenuated total reflection (Pt-ATR) accessory with a diamond crystal as the ATR element in the region 400–4,000 cm^−1^. The optical resolution of this spectrometer is 4 cm^−1^. All the spectra were recorded under vacuum using the double-side forward-backward acquisition mode.

### Fourier Transform-Raman Spectroscopy

The FT-Raman spectrum of CAF-CA cocrystal was recorded by using a MultiRAM spectrometer (Bruker) with a 1,064 nm laser line as the excitation line in the region 200–4,000 cm^−1^. A laser power of 500 mW was focused on the sample as spectral resolution of 4 cm^−1^ and about 512 scans were recorded.

## Computational and Theoretical Details

The density functional theory (DFT) methods (Geerlings et al., 2014) with Becke’s three parameters (Lee-Yang-Parr; B3LYP) (Lee et al., 1988; Becke, 1993; Parr, 1980) and standard 6–311++G (d, *p*) basis set (Andersson and Uvdal, 2005) were used to obtain the ground state optimized geometries of CAF, CA, monomer and dimer model of CAF-CA cocrystal by using Gaussian 09 program package (Frisch, 2009).

The binding energy (B.E.) of formation of monomer and dimer model of CAF-CA cocrystal can be calculated as:

$$B.E.=\left[E_{\mathrm{CAF}-\mathrm{CA}\;\text{model}}-\left(E_{\mathrm{CAF}}+E_{\mathrm{CA}}\right)\right]$$

Where, E_CAF-CA model_, E_CAF_ and E_CA_ are the ground state optimized energies of monomer or dimer model, CAF and CA, respectively.

The Raman activities produced by the DFT calculations can’t be used directly as Raman intensities. The Raman scattering cross section, ∂σ_j_/∂Ω which are proportional to Raman intensities, may be calculated from the Raman scattering amplitude and predicted wavenumbers for each normal mode using the relationship (Polavarapu et al., 1990)

$$\frac{\partial \sigma_{j}}{\partial \Omega \;}=\left(\frac{2^{4}\pi^{4}}{45}\right)\left(\frac{{\left(\nu_{0}-\nu_{j}\;\right)}^{4}}{1-\exp\left[\frac{-hc\nu_{j}}{kT}\right]}\right)\left(\frac{h}{8\pi^{2}c\upsilon_{j}}\right)S_{j}$$

Where, ν_0_ is the wavenumber of the Raman excitation line, ν_j_ is the predicted wavenumber, S_j_ is the Raman scattering activity of the *j*th normal mode and h, c, k are the universal constants.

Following the work of Pulay et al. (Pulay et al., 1979), a complete set of internal coordinates was defined and then the assignments of each normal mode were made on the basis of potential energy distribution (PED) analysis, calculated using Gar2Ped software package (Martin et al., 1995). For the pictorial representation of titled molecules and to obtain the calculated data GaussView program (Frisch et al., 2000) was used. The NBO calculations were performed using the same Gaussian 09 program and B3LYP/6–311++G (d,*p*) basis set. The hyperconjugative interaction energy (stabilization energy, E^(2)^) was estimated from second-order perturbation approach (Reed et al., 1988).

Geometrical and topological parameters for bonds of interacting atoms and their molecular graph have been obtained by using AIM2000 software (Bader and Cheeseman, 2000). Espinosa (Espinosa et al., 1998) correlated the proportionality between hydrogen bond energy (E_HB_) and potential energy density (V_BCP_) at H∙∙∙O contact by the expression, E_HB_ = 0.5 V_BCP_. The global quantum chemical indices; electronegativity (χ), chemical potential (μ), hardness (η), electrophilicity index (ω) and softness (S) are obtained by energies of HOMO, LUMO (E_HOMO_, E_LUMO_) and can be calculated by the equations as (Parr and Pearson, 1983; Geerlings et al., 2003; Chattaraj and Roy, 2007),

$$\chi =\;-\frac{1}{2}\left(E_{HOMO}+\;E_{LUMO}\right)$$

$$\mu =\;-\chi =\;\frac{1}{2}\left(E_{HOMO}+E_{LUMO}\right)$$

$$\eta =\;\frac{1}{2}\left(E_{LUMO}-E_{HOMO}\right)$$

$$S=\;\frac{1}{2\eta }$$

$$\omega =\;\frac{\mu^{2}}{2\eta }$$

The energy of stabilization can be discussed in terms of electrophilicity index (ω), whenever the given system receives an additional electronic charge (∆N) from its surroundings. The maximum electronic charge (∆N_max_) that any electrophile may accept from the surroundings may be represented as:

$$\Delta \;N_{\max}=\;-\frac{\mu }{\eta }$$

The electrophilic charge transfer (ECT) (Pearson, 1989) i.e. the amount of charge transfer between two interacting molecules P and Q may be calculated as:

$$ECT={\left(\Delta N_{\max}\right)}_{P}-{\left(\Delta N_{\max}\right)}_{Q}$$

Where, ${\left(\Delta N_{\max}\right)}_{P}=\;-\frac{\mu_{P}}{\eta_{P}}$ and. ${\left(\Delta N_{\max}\right)}_{Q}=\;-\frac{\mu_{Q}}{\eta_{Q}}$

Molar refractivity (MR) is a parameter defining the quantitative structure-activity relationship (QSAR) (Sawale et al., 2016) of a drug molecule. It is well defined using Lorentz-Lorenz formula (Verma and Hansch, 2005) and can be calculated as:

$$MR=\left[\frac{n^{2}-1}{n^{2}+2}\right]\left(\frac{MW}{\rho }\right)=1.333\pi N\alpha$$

Where, n is the refractive index, MW is the molecular weight, ρ is the density, (MW/ρ) is the molar volume, α is the polarizability and N is the Avogadro’s number of a molecule.

## Results and Discussion

### Geometry Optimization and Energies

**FIGURE 2:**
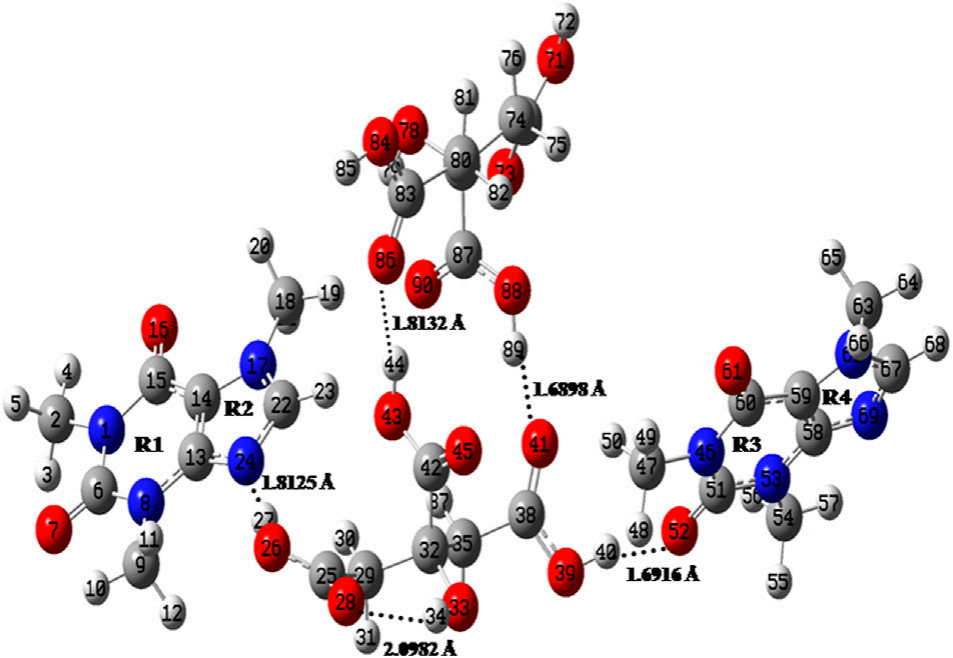
Optimized structure for dimer model of cocrystal with the atomic numbering scheme adopted in the present study.

The initial crystallographic data of monomer, dimer model of cocrystal and that of CAF and CA were taken from their reported crystal structures (Churakov, 2007; Lehmann and Stowasser, 2007; Smit and Hagen, 2015), respectively. The ground state optimized structures of CAF, CA, monomer and dimer model of CAF-CA cocrystal are shown in Supplementary Figures S4, S5, S6 and Figure 2, respectively.

The optimized structural parameters (bond lengths, bond angles and dihedral angles) of CAF with both monomer and dimer model of CAF-CA cocrystal along with their respective experimental values (Lehmann and Stowasser, 2007; Smit and Hagen, 2015) has been listed in Supplementary Table S1.

A comparison between the optimized geometrical parameters of CAF and monomer model of cocrystal showed that the differences in the values of bond lengths were not more than 0.005 Å. The majorities are among the length of bonds namely, N17-C22, C22-N24 and C13-N24 of imidazole ring R2, having values 1.3350/1.3471 Å, 1.3287/1.3346 Å and 1.3572/1.3635 Å, in CAF/monomer model, respectively. These differences are attributed to the presence of strong intermolecular hydrogen bonding (O26-H27∙∙∙N24) between hydroxyl (O26-H27) group of CA and N24 atom of imidazole ring of CAF, as shown in Supplementary Figure S6. Also, the corresponding changes were noticed in the bond angles and dihedral angles of the above bonds.

The optimized geometrical parameters of monomer/dimer model of cocrystal were also compared with the experimental values (Smit and Hagen, 2015) and it was found that the computed values of dimer of cocrystal is closer to the observed results than monomer model, due to incorporation of intermolecular hydrogen bond interactions, as shown in Supplementary Table S1 and Supplementary Figure S3. The computed bond lengths, bond angles and dihedral angles values of monomer/dimer model of cocrystal show the replication with the experimental data of 0.0366/0.0348 Å, 1.9/0.1° and 10.7/7.5°, respectively, except for the bonds and angles with one atom as hydrogen.

The ground state optimized energies of CAF, CA, monomer and dimer model of cocrystal are obtained to be −427,060.4516, −477,105.3403, −904,169.1360, and −1808356.6764 kcal mol^−1^, respectively. The values of B.E. of monomer and dimer model of CAF-CA cocrystal were calculated to be −3.3441 and −25.0926 kcal mol^−1^, respectively. It has been corrected due to the basis set superposition error (BSSE) by means of the standard counterpoise method (Runge and Gross, 1984) and found to be −2.4405 and −21.4948 kcal mol^−1^, respectively.

### Vibrational Assignment

The crystal structure analysis demonstrated that CAF, CA, monomer and dimer model of cocrystal consists of 24, 21, 45, and 90 atoms; hence undergo 66, 57, 129, and 264 modes of vibrations, respectively. All fundamental vibrations are active in both Raman scattering and IR absorption. The complete vibrational assignments of CAF and CA are given in Supplementary Tables S2, S3, respectively. The combined PED assignments of monomer and dimer model of cocrystal along with experimental values are presented in Supplementary Table S4. The discrepancies between the calculated and experimental vibrational wavenumbers were because of performing the calculations on isolated molecule and in the absence of anharmonicity. The linear scaling procedure (WLS) (Yoshida et al., 2002) is used to give the best fit to the experimental vibrational spectra by the following formula:

$$\left[\nu_{obs}=\left(1.0087-0.0000163\;\upsilon_{cal}\right)\;\upsilon_{cal}\right]cm^{-1}$$

**FIGURE 3:**
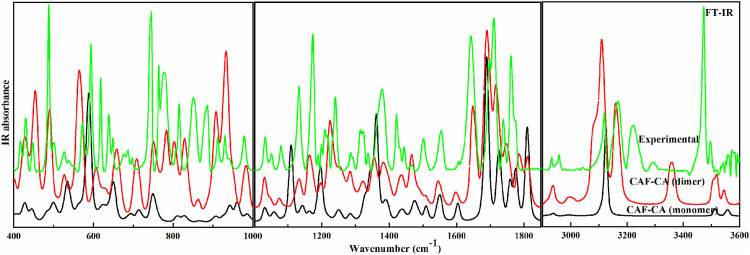
Experimental and calculated (scaled) IR absorbance spectra of monomer and dimer model of cocrystal in the region 400–1,000 cm^−1^, 1,001–1850 cm^−1^ and 2,900–3,600 cm^−1^.

**FIGURE 4:**
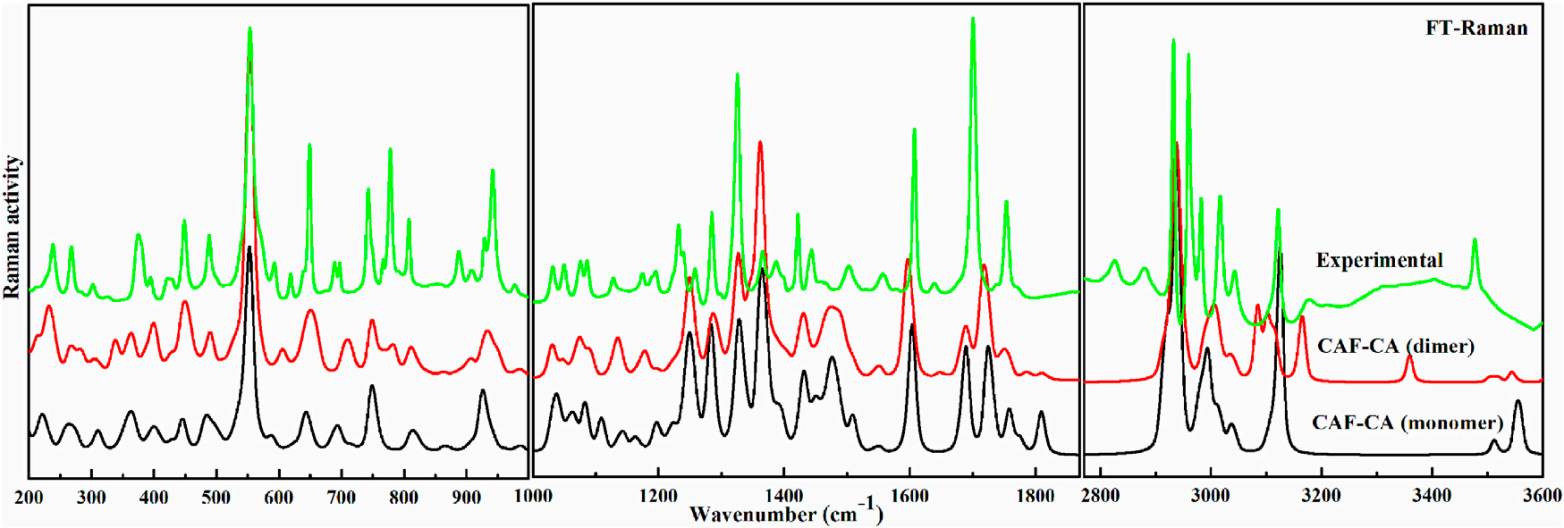
Experimental and calculated (scaled) Raman scattering spectra of monomer and dimer model of cocrystal in the region 200–999 cm^−1^, 1,000–1870 cm^−1^ and 2,770–3,600 cm^−1^.

The descriptions of the modes involved in hydrogen bonding along with bond length and stretching frequency are discussed in Table 1. Table 1 clearly indicates that theoretically obtained vibrational wavenumbers and bond length values in dimer matches well with the observed values, since it incorporates all the nearest possible hydrogen bonding interactions that are not present in the case of monomer of CAF-CA cocrystal. The comparative experimental and simulated FT-IR, FT-Raman spectra of CAF and CA are shown in Supplementary Figures S7–S10, respectively. While the comparison of observed and calculated (scaled) IR and Raman spectra of monomer with dimer model of CAF-CA cocrystal are shown in Figures 3, 4, respectively.

**TABLE 1:** Experimental and theoretical bond length (Å) and stretching frequency (cm^−1^) of the bonds involved in hydrogen bonding.

Groups present in CAF					Groups present in CA			
	C-N in imidazole ring		C=O group of pyrimidine ring		C=O group		O-H group	
Molecules	Bond length	Stretching frequency	Bond length	Stretching frequency	Bond length	Stretching frequency	Bond length	Stretching frequency
Experimental								
CAF	1.3281	1325,1329 (IR, Raman)	1.2226	1696,1699 (IR, Raman)	-	-	-	-
CA	-	-	-	-	1.2151	1744,1737 (IR, Raman)	0.8642	3,224 (IR)
							0.9097	3286,3291 (IR, Raman)
							0.8823	3286,3291 (IR, Raman)
							0.8923	3493,3496 (IR, Raman)
CAF-CA cocrystal	1.3416	1323,1325 (IR, Raman)	1.2397	1642,1639 (IR, Raman)	1.2150	1,697,1700 (IR, Raman)	0.7940	3120,3121 (IR, Raman)
							0.8876	3077,3079 (IR, Raman)
							0.9801	3,168, 3,177 (IR, Raman)
							0.9581	3,295 (IR)
Theoretical								
CAF	1.3287	1,335 (C14-N6)	1.2181	1719(C4 = O10)	-	-	-	-
CA	-	-	-	-	1.2045	1776 (C8 = O2)	0.9711	3,507 (O7-H19)
							0.9699	3,553 (O6-H20)
							0.9699	3,553 (O1-H21)
							0.9692	3,565 (O4-H18)
monomer Model of cocrystal	1.3346	1,330(C22-N24)	1.2152-	1727(C6 = O7)-	1.2045	1776 (C38 = O41)	0.9711	3,513 (O33-H34)
							0.9919	3,124 (O26-H27)
							0.9699	3,554 (O39-H40)
							0.9693	3,559 (O43-H44)
dimer Model of cocrystal	1.3388	1,323(C22-N24)	1.2170, 1.2309	1719 (C6 = O7), 1,646 (C51 = O52)	1.2256, 1.1975	1,689 (C38 = O41, 1809 (C70 = O73)	0.9703, 0.9713	3,511 (O78-H79)
							0.9945	3,499 (O33-H34)
							0.9707	3,078 (O26-H27)
							0.9929, 0.9647	3,538 (O84-H85)
							0.9812, 0.9950	3,157 (O39-H40)
								3,602 (O71-H72)
								3,352 (O43-H44)
								3,108 (O88-H89)

#### Discussions of Modes Involved in Hydrogen Bonding

##### Carbonyl (C=O) Group of CAF

The C=O group (acts as hydrogen bond acceptor) easily interacts with hydrogen bond donor molecule like CA and generally have strong absorption band in the region of 1850–1,550 cm^−1^ (Socrates, 2004). In the CAF, monomer no C=O group is involved in the intermolecular hydrogen bonding and in case of dimer model, one of the C=O group of CAF and O-H group of CA is forming hydrogen bond with each other as shown in Supplementary Figures S2, S4, S6, respectively. The stretching vibrations of one of the C=O group in CAF (C4 = O10), monomer (C6 = O7) and dimer model (C51 = O52) of cocrystal are calculated at 1719, 1727, and 1,646 cm^−1^, respectively that was observed at 1,642/1,639 cm^−1^ in IR/Raman spectra of CAF-CA cocrystal. It clearly indicates the red shifting in calculated wavenumbers of 73 cm^−1^ and 81 cm^−1^ with respective increments in bond lengths of 0.0128 Å and 0.0157 Å of dimer than CAF and monomer model, respectively. This is due to the presence of intermolecular hydrogen bonding (C51 = O52∙∙∙H40-O39) in dimer model (Figure 2) confirming the cocrystal formation. From Table 1, it is also clear that the calculated bond lengths and wavenumber values in dimer correlate well with the experimental data than CAF and monomer model of cocrystal.

##### C-N of Imidazole Ring of CAF

The identification of C-N stretching vibrations is difficult task, as these are mixed with several other bands (Socrates, 2004). In the CAF molecule no C-N group is involved in the intermolecular hydrogen bonding, however, one of the C-N mode (C22-N24) of CAF and hydroxyl group (O26-H27) of CA is involved in the hydrogen bonding with each other in monomer and dimer model, as given in Supplementary Figures S2, S4, S6, respectively. The C-N stretching vibration of imidazole ring R2 of CAF (C14-N6), monomer (C22-N24) and dimer (C22-N24) model is calculated at 1,335, 1,330, and 1,323 cm^−1^, respectively, corresponding to the observed peak at 1,323/1,325 cm^−1^ in IR/Raman spectra in cocrystal. The monomer and dimer model of cocrystal are closer to the experimental values due to the incorporation of intermolecular hydrogen bonding (C22-N24∙∙∙H27-O26). The bond distance of N24-H27 bond in dimer (1.8125 Å) is greater (due to the attachment of other two neighboring molecules as depicted in Figure 2) than monomer.

##### Carbonyl (C=O) Group Vibrations of CA

Three carboxyl groups are present in CA molecule and usually observed in 1720–1,680 cm^−1^ region (Socrates, 2004). In CA and monomer, all C=O groups are free while in dimer one of the C=O and OH groups of CA are involved in the intermolecular hydrogen bonding with each other as shown in Supplementary Figures S2, S5, S6, respectively. From Supplementary Table S3, it is clear that the stretching vibrations of one of the C=O group in CA (C8 = O2), monomer (C38 = O41) and dimer (C38 = O41) model are calculated at 1776 cm^−1^, 1776 cm^−1^ and 1,689 cm^−1^ corresponding to the observed peak at 1,697/1700 cm^−1^ in IR/Raman spectra in CAF-CA cocrystal. The downward shifting and increment in calculated stretching wavenumber and bond length of (C38 = O41) group by 87 cm^−1^/87 cm^−1^ and (0.0211 Å)/(0.0211 Å), respectively of dimer than CA/monomer model of cocrystal confirms the presence of intermolecular hydrogen bonding (C38 = O41∙∙∙H89-O88).

##### Hydroxyl (O-H) Groups of CA

Four O-H groups are present in CA, three are present in carboxyl group and one is free. In CA all are independent, while in monomer and dimer one and all of the O-H, respectively of CO-OH group is involved in the intermolecular hydrogen bonding as given in Supplementary Figures S2, S5, S6, respectively. The stretching vibrations of one of the O-H group in CA (O6-H20), monomer (O26-H27) and dimer (O26-H27) model of cocrystal are calculated at 3,553, 3,124, and 3,078 cm^−1^, respectively corresponding to the observed values at 3,077/3,079 cm^−1^ in IR/Raman spectra of cocrystal. It clearly indicates the downward shifting and respective increment in calculated stretching wavenumbers and bond lengths by 429 cm^−1^/475 cm^−1^ and 0.022 Å/0.0246 Å of monomer/dimer model, respectively than CA. Hence this group is involved in intermolecular hydrogen bonding such as (O26-H27∙∙∙N24-C22).

Also, in CA, monomer and dimer model alcoholic (O33-H34) group is forming intramolecular hydrogen bonding with carbonyl (C25 = O28) group as shown in Supplementary Figures S2, S5, S6. It is calculated at 3,507 cm^−1^/3,513 cm^−1^/3,499 cm^−1^ and observed at 3,120 cm^−1^ in the IR and 3,121 cm^−1^ in Raman spectra of cocrystal, respectively. Thus, from the above analysis it is clear that the calculated wavenumbers of dimer have a good resemblance to the experimental values than isolated (CAF or CA) and monomer model of cocrystal due to the incorporation of hydrogen bonding interactions.

### Powder X-Ray Diffraction

Powder X-ray diffraction (PXRD) measures the diffraction pattern of crystalline material and it is widely used to study crystal structures. Each crystalline material produces a specific pattern depending on the structure of its crystal lattice. In other words, it plays an important role in the determination or tracking of any change in the solid structure (Malik et al., 2020). In order to evaluate the obtained cocrystal form, we have compared the measured diffraction pattern with the one simulated from the crystal structure (Smit and Hagen, 2015) and it gives a near perfect match as shown in Supplementary Figure S1.

### Differential Scanning Calorimetry (DSC)

DSC technique (Al-Mutabagani et al., 2020) was used as a complementary tool with PXRD. The DSC data of CAF-CA cocrystal is shown in Figure S2. Data analysis indicates that the endothermic (melting phenomenon) onset is observed at 159.7°C (peak: 160.8°C; heat of fusion: 141.8 J/g). CAF has a higher melting point (235°C) compared to the cocrystal and CA (150°C) and obtained data is in line with the previous report (Smit and Hagen, 2015).

### Topological and Energy Parameters at Bond Critical Points

Bader proposed the quantum theory of atoms in molecules (QTAIM) and defined the parameters at bond critical point (BCP; at which electron density is minimum) (Bader, 1985). Geometrical and topological parameters like electron density (ρ_BCP_) and the Laplacian of the electron density (∇^2^_BCP_) at the BCPs are used to characterize the strength of hydrogen bonds in molecules (Bader, 2011). According to Koch and Popelier criteria (Koch and Popelier, 1995), an existence of hydrogen bond follows BCP for donor proton (H)∙∙∙acceptor (A)’, for which the ρ_BCP_ lies within the range 0.002–0.040 a. u. and (∇^2^ρ_BCP_) is in the range of 0.024–0.139 a. u. Further Rozas et al. (Rozas et al., 2000) describes the characterization of hydrogen bond interactions in molecules as follows: 1) For strong and covalent nature: (∇^2^ρ_BCP_) < 0 and H_BCP_ < 0; 2) For medium and partially covalent nature: (∇^2^ρ_BCP_) > 0 and H_BCP_ < 0; 3) For weak and electrostatic nature: (∇^2^ρ_BCP_) > 0 and H_BCP_ > 0; 4) The bond distance of two interacting atoms is smaller than the sum of van der Waals radii of these atoms. Desiraju (Desiraju, 2000) outlines the concept of conventional (strong) and non-conventional (weak) hydrogen bonds, and proposed that C-H∙∙∙O interaction could also be relevant in drug design strategies and stabilization of molecule.

**FIGURE 5:**
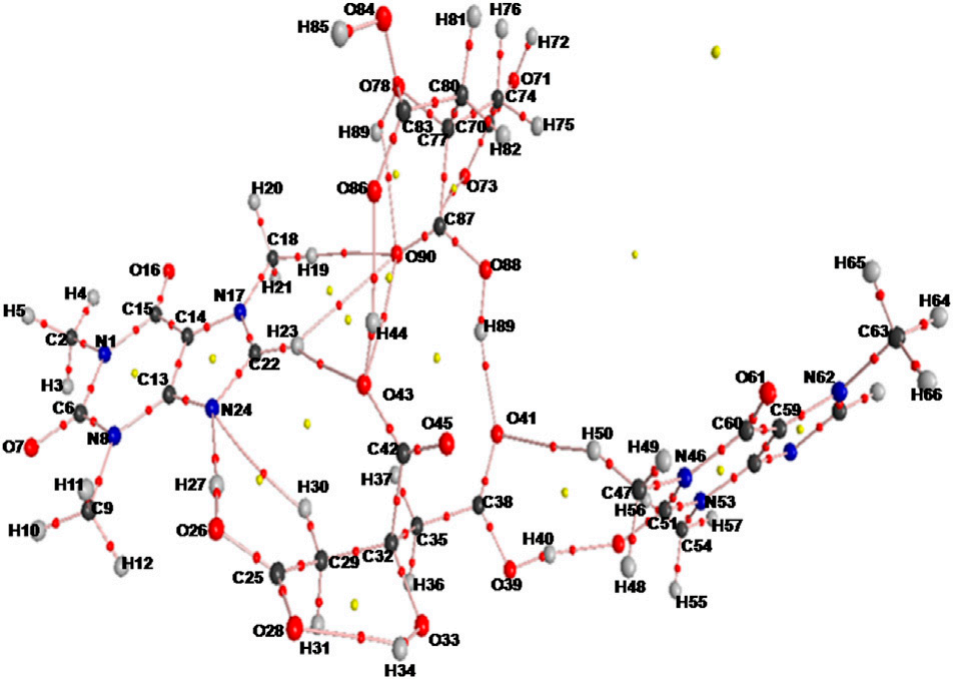
Molecular graph of dimer model of cocrystal: BCPs (small red spheres), ring critical points (small yellow sphere), bond paths (pink lines).

The molecular graphs of monomer and dimer model of cocrystal are shown in Figure 5, Supplementary Figure S11, respectively. All calculated geometrical and topological parameters for intra- and intermolecular hydrogen bonding interactions of monomer and dimer model of cocrystal are listed in Supplementary Tables S2, S5, respectively. Supplementary Tables S6, S7, represent the geometrical criteria for the existence of hydrogen bonds in monomer and dimer model of cocrystal, respectively. On the basis of these parameters given in Supplementary Table S5, in monomer model of cocrystal, O28∙∙∙H34 (2.0543 Å) and N24∙∙∙H27 (1.8038 Å) are medium and partially covalent intra- and intermolecular hydrogen bonds, respectively, as (∇^2^ρ_BCP_) > 0 and H_BCP_ < 0. In dimer model of cocrystal, the categorization of hydrogen bonds on the basis of E_HB_ is in following order: O88-H89∙∙∙O41 > O39-H40···O52 > O26-H27···N24 > O43-H44···O86 > O78-H79···O90 > O33-H34···O28 > C22-H23···O43 > C29-H30∙∙∙N24 > C47-H50∙∙∙O41 > C18-H19∙∙∙O90 > C22-H23∙∙∙O90 and showed medium and partially covalent nature on the basis of criteria, (∇^2^ρ_BCP_) > 0 and H_BCP_ < 0 (as depicted in Table 2). Based on this Desiraju criteria (Desiraju, 2000); O88-H89∙∙∙O41, O39-H40···O52, O26-H27···N24, O43-H44···O86, O78-H79···O90 and O33-H34···O28 are conventional, while C22-H23···O43, C29-H30···N24, C47-H50···O41 and C18-H19···O90 are non-conventional hydrogen bonds.

**TABLE 2:** Geometrical parameter (bond-length) and topological parameters for bonds of interacting atoms of intra- and intermolecular hydrogen bonding interactions of dimer model of cocrystal: electron density (ρ_BCP_), Laplacian of electron density (∇^2^ρ_BCP_), electron kinetic energy density (G_BCP_), electron potential energy density (V_BCP_), total electron energy density (H_BCP_) at bond critical point (BCP) and H-bond energy (E_HB_).

Interactions	Bond length (Å)	ρ_BCP_ (a.u.)	∇^2^ρ_BCP_ (a.u.)	G_BCP_ (a.u.)	V_BCP_ (a.u.)	H_BCP_ (a.u.)	E_HB_ (kcal mol^−1^)
O88-H89∙∙∙O41	1.6898	0.0418	_0.1404_	0.0022	−0.0396	−0.0374	−12.4247
O39-H40∙∙∙O52	1.6916	0.0409	_0.1416_	0.0017	−0.0388	−0.0371	−12.1737
O26-H27∙∙∙N24	1.8125	0.0402	_0.0975_	0.0040	−0.0325	−0.0285	−10.1970
O43-H44∙∙∙O86	1.8132	0.0293	_0.1164_	−0.0,022	−0.0248	−0.027	−7.7811
O78-H79∙∙∙O90	2.0515	0.0243	_0.1006_	−0.0026	−0.0199	−0.0225	−6.2437
O33-H34∙∙∙O28	2.0982	0.0202	_0.0684_	−0.0013	−0.0145	−0.0158	−4.5494
C22-H23∙∙∙O43	2.3402	0.0110	_0.0388_	−0.0016	−0.0066	−0.0082	−2.0708
C29-H30∙∙∙N24	2.6221	0.0087	_0.0273_	−0.0010	−0.0048	−0.0058	−1.5060
C47-H50∙∙∙O41	2.5521	0.0078	_0.0239_	−0.0007	−0.0045	−0.0052	−1.4119
C18-H19∙∙∙O90	2.5247	0.0072	_0.0242_	−0.0009	−0.0042	−0.0051	−1.3178
C22-H23∙∙∙O90	2.8593	0.0035	_0.0132_	−0.0006	−0.0020	−0.0026	−0.6275

The intra- and intermolecular C70 = O73···C87 and C42-O43···O90 interactions in the dimer model of cocrystal are listed in Supplementary Table S8. Allen et al. (Allen et al., 1998) described the condition of preferred bonding geometry with C···O distance <3.6 Å and C=O···C angle between the range of 80˚–100°. From Supplementary Table S8, it is clear that C70 = O73···C87 satisfies above described criteria (as bond distance = 2.8275 Å < 3.6 Å and bond angle 89.0108°) with interaction energy −2.5728 kcal mol^−1^. Also, the C42-O43?O90 bond is formed due to the H44 atom which is involved in formation of strong hydrogen bonding interaction (O43-H44∙∙∙O86) and plays significant role in the interaction of two oxygen atoms by bringing them closer, its energy of interaction is found to be −1.2864 kcal mol^−1^.

### Natural Bond Orbital Analysis

The natural bond orbital (NBO) analysis is one of the reliable tools used for examining the conjugative interactions or charge transfer phenomenon. It provides an important basis for evaluating intra- and intermolecular bonding in the polyatomic molecules (Jauhar et al., 2018). The intensity of interaction between electron donors and electron acceptors i.e. donating tendency depends on higher value of stabilization energy E^(2)^. Delocalization of electron density between occupied Lewis type (bond or lone pair) NBOs and formally unoccupied (antibond or Rydberg) non-Lewis type NBOs correspond to a stabilized donor-acceptor interactions. NBO analysis also confirms the hyperconjugative charge transfer interactions from filled lone pairs (n) to the unfilled anti-bond σ*, (n → σ*) in the hydrogen bonded system (Dunnington and Schmidt, 2012).

In the present work, utilizing the effect of the second-order micro-disturbance theory of the Fock Matrix (Reed et al., 1988), all nearest possible intra- and intermolecular interactions have been defined for monomer and dimer model of cocrystal and are given in Supplementary Tables S9, S10, respectively. From Supplementary Table S9, it can be seen that in monomer model of cocrystal, the intramolecular interaction in unit 1 (CAF) and 2 (CA) named [n (1)N17 → π*(C22-N24)] and [n (2)O26 → π*(C25 = O28)] with maximum E^(2)^ of 65.68 kcal mol^−1^ and 46.14 kcal mol^−1^ leading to the stability of ring R2 and molecule, respectively. The charge transfer interaction from unit 1 (CAF) to unit 2 (CA) [n (1)N24 → σ*(O26-H27)] with E^(2)^ of 23.27 kcal mol^−1^ directing the formation of strong intermolecular hydrogen bonding interaction (N24∙∙∙H27-O26) in monomer model of cocrystal.

From Supplementary Table S10, it is clear that in dimer model of cocrystal, the charge transfer interactions in between units; 1 (CAF) to 2 (CA), 2 (CA) to 1 (CAF), 2 (CA) to 3 (CAF), 2 (CA) to 4 (CA), 3 (CAF) to 2 (CA), 3 (CAF) to 4 (CA), 4 (CA) to 1 (CAF), 4 (CA) to 2 (CA) and 4 (CA) to 3 (CAF); such as [n (1)N24 → σ*(O26-H27)] [n (1)O43 → σ*(C22-H23)] [π(C38 = O41) → σ*(C47-H50)] [n (2)O41 → σ*(O88-H89)] [n (1)O52 → σ*(O39-H40)] [n (1)O52 → σ*(O84-H85)] [n (1)O90→ σ*(C18-H19)] [n (1)O86 → σ*(O43-H44)] and [σ(C87 = O90)→ σ*(N46-C47)] with the E^(2)^ of 23.64, 2.00, 0.32, 12.35, 13.23, 0.08, 0.72, 8.34, and 0.18 kcal mol^−1^, respectively, results in intermolecular hydrogen bonding interaction (N24∙∙∙H27-O26, O43∙∙∙H23-C22, O41∙∙∙H89-O88, O52∙∙∙H40-O39, O52∙∙∙H85-O84, O90∙∙∙H19-C18, and O86∙∙∙H44-O43) causing stabilization and formation of CAF-CA cocrystal. Also, there occur strong intramolecular charge transfer interactions within unit 1 (CAF), unit 2 (CA), unit 3 (CAF) and unit 4 (CA) [n (1)N17 → π*(C22-N24)] [n (2)O39 → π*(C38 = O41)] [π(C51 = O52) → π*(N46-C51)] and [n (2)O88 → π*(C87 = O90)] with E^(2)^ of 66.72, 59.85, 128.08, and 54.51 kcal mol^−1^, respectively which provide extra stability to the cocrystal, as shown in Figure 2.

### Frontier Molecular Orbital Analysis

**FIGURE 6:**
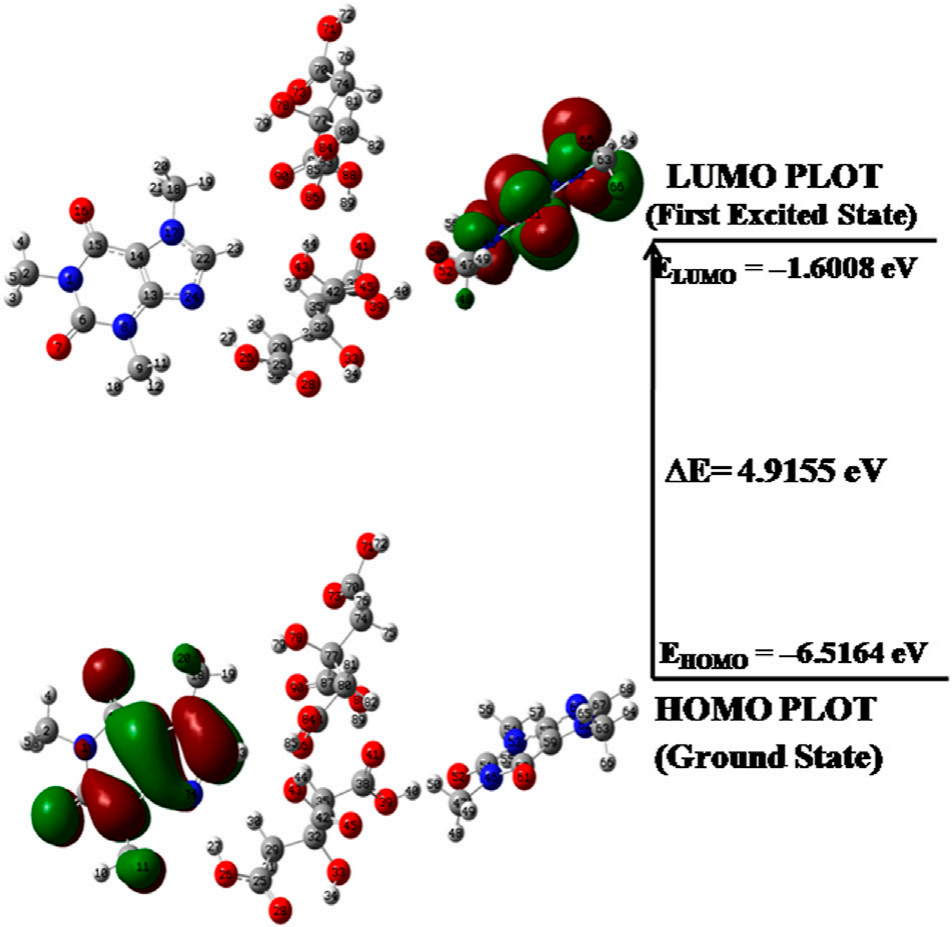
HOMO-LUMO plot of dimer model of cocrystal with orbital involved in electronic transitions.

According to orbital symmetry concepts, the two types of orbitals i.e. highest occupied molecular orbital (HOMO) and lowest unoccupied molecular orbital (LUMO) orbitals and its energy gap $\Delta \text{E}=\left({\text{E}}_{\text{LUMO}}-{\text{E}}_{\text{HOMO}}\right)$ are responsible for chemical stability and reactivity of a molecule (Ghosh and Jana, 1999; Ucak-Astarlioglu et al., 2017). The HOMO act as an electron donor and its energy (E_HOMO_) is directly related to ionization potential and the LUMO act an electron acceptor and its energy (E_LUMO_) is directly related to electron affinity. A small gap implies low stability, high polarizablility and is generally associated with high chemical reactivity (Shukla et al., 2017) and vice versa. The electronic transition features of HOMO-LUMO orbitals of CAF, CA, monomer and dimer model of cocrystal are given in Supplementary Figures S12–S14 and Figure 6, respectively and their energy gaps are found to be 5.0320, 7.2663, 5.0377 and 4.9155 eV, respectively. So, it is clear that dimer and hence, CAF-CA cocrystal is more reactive, less stable than monomer model and CAF.

### Chemical Reactivity Descriptors

Global reactivity descriptors play an important role in predicting chemical reactivity of a molecular system, whereas the local reactivity parameters are useful to analyze the site selectivity of a chemical system. They also help in the prediction of the electrophilic and nucleophilic behavior of the molecule.

#### Global Reactivity Descriptors

The global reactivity descriptors such as electronegativity (χ), chemical potential (μ), hardness (η), electrophilicity index (ω) and softness (S) computed by means of energies of E_HOMO_, E_LUMO_ for CAF, CA, monomer and dimer model of cocrystal are listed in Table 3 η and S, parameters are reciprocal to each other and describe the chemical reactivity and stability of a molecule (Olatunbosun and Banjo, 2013). According to Parr et al. (Parr and Pearson, 1983), ω measures the propensity or capacity of a species to accept electrons and predicts stabilization in energy when system gains additional charge ΔN from surrounding.

**TABLE 3:** Calculated E_HOMO_, E_LUMO_, energy gap (E_L_-E_H_), chemical potential (μ), electro negativity (χ), global hardness (η), global softness (S) and global electrophilicity index (ω) at 298.15 K for CAF, CA, monomer model and dimer model of cocrystal.

Molecule	E_H_ (eV)	E_L_ (eV)	E_L_-E_H_ (eV)	χ (eV)	μ (eV)	η (eV)	S (eV)	ω (eV)	ΔN_max_
CAF	−6.3452	−1.3132	5.0320	3.8292	−3.8292	2.5160	0.1987	2.9139	1.5219
CA	−8.1297	−0.8634	7.2663	4.4966	−4.4966	3.6332	0.1376	2.7827	1.2376
CAF-CA (monomer)	−6.9828	−1.9451	5.0377	4.4640	−4.4640	2.5189	0.1985	3.9556	1.7722
CAF-CA (dimer)	−6.5164	−1.6008	4.9155	4.0586	−4.0586	2.4578	0.2034	3.3510	1.6513

The high values of ω and μ for CAF and low values of ω and μ for CA favor their electrophilic and nucleophilic behavior, respectively. Since the values of S of dimer > CAF > monomer > CA, it means that dimer model and hence cocrystal is softer than CAF. The electrophilic charge transfer (ECT) is calculated to be 0.2843 eV and is positive, which indicates that charge flows from CA (coformer) to CAF (API) in the cocrystal. Therefore, CA acts as electron donor and CAF acts as an electron acceptor.

#### Local Reactivity Descriptors

Fukui function f(r) (Li and Evans, 1995) is an approximate local reactivity descriptor which is more reactive center in chemical species. By using Hirshfeld atomic charges three kinds of condensed Fukui functions (${\text{f}}_{\text{k}}^{+},{\text{f}}_{\text{k}}^{-},{\text{f}}_{\text{k}}^{0}$), local softness (${\text{s}}_{\text{k}}^{+},{\text{s}}_{\text{k}}^{-},{\text{s}}_{\text{k}}^{0}$) and electrophilicity indices (${\text{ω}}_{\text{k}}^{+},{\text{ω}}_{\text{k}}^{-},{\text{ω}}_{\text{k}}^{0}$) for nucleophilic, electrophilic and radical attacks can be defined, which are capable to differentiate between the reactive atomic centers. The calculated values of local reactivity descriptors for monomer and dimer model of cocrystal are shown in Supplementary Tables S11, S12, respectively. From Supplementary Table S11, it is clear that the maximum values of all three descriptors (${\text{f}}_{\text{k}}^{+},{\text{s}}_{\text{k}}^{+},{\text{ω}}_{\text{k}}^{+}$) and (${\text{f}}_{\text{k}}^{-},{\text{s}}_{\text{k}}^{-},{\text{ω}}_{\text{k}}^{-}$) at O7 and C22 atoms indicate that these atomic centers are more favourable for nucleophilic and electrophilic attack, respectively in monomer model of cocrystal. While in dimer model of cocrystal, the maximum values of (${\text{f}}_{\text{k}}^{+},{\text{s}}_{\text{k}}^{+},{\text{ω}}_{\text{k}}^{+}$) and (${\text{f}}_{\text{k}}^{-},{\text{s}}_{\text{k}}^{-},{\text{ω}}_{\text{k}}^{-}$) are for O90 and H10 atoms, respectively, indicating these regions as favourable for nucleophilic and electrophilic attack, respectively.

### Molecular Electrostatic Potential Surface

**FIGURE 7:**
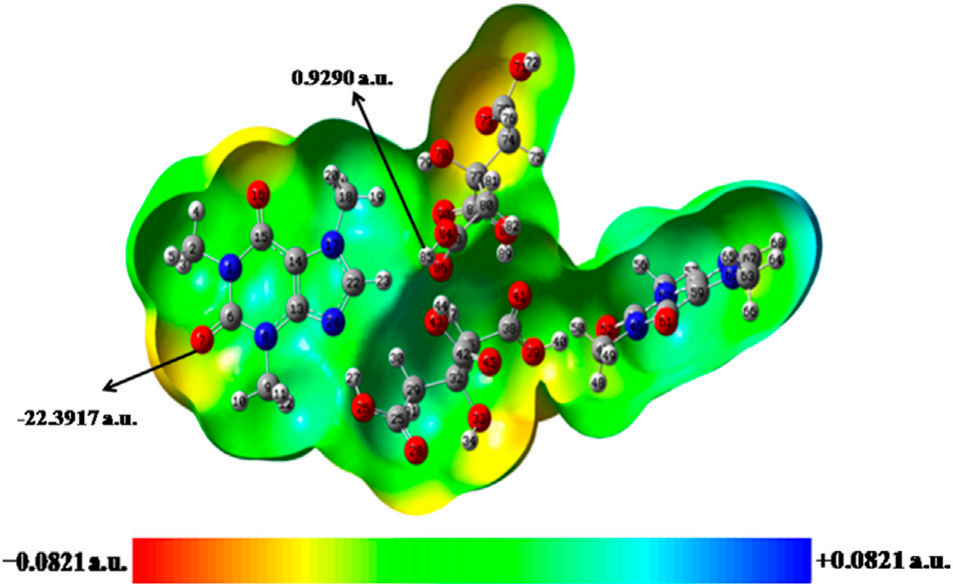
Molecular electrostatic potential (MESP) formed by mapping of total density over electrostatic potential in the gas phase for dimer model of cocrystal.

MESP (Perera et al., 2016) topography serves as a useful tool for predicting and understanding the interactive behavior of the molecular system. It quantifies the electron-rich and electron-deficient character of that region. Additionally, this well-established tool is efficient in understanding various chemical properties like chemical reactivity, intermolecular interactions and inductive effects, etc. (Dargent et al., 2015). The most negative (V_min_, red region) and positive (V_max_, blue region) electrostatic potential value in MESP indicate the electron donating and accepting tendency in a particular molecule, respectively (Matta, 2014). The MESP plots of CAF, CA, monomer and dimer model of cocrystal are given in Supplementary Figures S15–S17 and Figure 7, respectively.

The MESP isosurface of CAF depicts that red (negative potential) region is over carbonyl (C4 = O10) group of pyrimidine ring and N atom of imidazole ring, hence suitable for electrophilic attack (relative abundance of electrons). While blue region is distributed over methyl (C13H_3_) group of imidazole ring, hence suitable for nucleophilic attack (relative absence of electrons) as shown in Supplementary Figure S15. From the MESP of CA, the three carbonyl (C12 = O5, C8 = O2 and C13 = O3) and all the hydroxyl (O6-H20, O4-H18 and O1-H21) groups are suitable for electrophilic and nucleophilic attack, respectively. In monomer model of cocrystal, the electrophilic reactivity of N24 atom in CAF and nucleophilic reactivity of (O26-H27) group of CA are neutralized, due to the strong intermolecular interaction (N24?H27-O26) as shown in Supplementary Figure S17.

In case of dimer model of cocrystal, the nucleophilic and electrophilic reactivity of methyl (C18H_3_) and (C51 = O52) groups of CAF neutralizes due to the effect of intermolecular hydrogen bond interactions with neighboring molecule, respectively (as shown in Figure 7). Also, the electrophilic reactivity of (C38 = O41) group and nucleophilic reactivity of (O26-H27, O39-H40, O43-H44) groups of CA neutralize after the cocrystal formation. The V_max_ and V_min_ values for dimer model of cocrystal are for H85 atom of hydroxyl group and O7 atom of carbonyl group and are found to be 0.9290 a. u. and −22.3917 a. u., respectively. These atoms indicate the propensity toward hydrogen bond formation in the crystal structure of cocrystal.

### Molar Refractivity

Lipinski et al. (Lipinski et al., 1997) proposed a set of rules, following which it would be easy to show drug-like properties for a compound and is known as “Rule of five”. According to this rule, the compound must have 1) molar refractivity (MR) value ranging from 40 to 130 e. s.u., 2) the molecular weight within range 180–500 g mol^−1^, 3) the number of atoms from 20–70. Further, the MR value is responsible for the lipophilicity, binding property, polarizability and dispersitivity of the molecular system (Magyari et al., 2018). The calculated values of MR for CAF, monomer and dimer model of cocrystal are 30.26 e. s.u., 66.69 e. s.u. and 105.70 e. s.u., respectively. The molecular weight of CAF and CAF-CA cocrystal are 194.19 g mol^−1^ and 386.31 g mol^−1^, respectively. Also, the numbers of atoms in CAF and monomer model of cocrystal are 24 and 45, respectively. Hence, corresponding to the Lipinski’s rule CAF-CA cocrystal obeys the criteria of drug-likeness and hence is suitable for orally active form as drug.

## Conclusion

The experimental and theoretical calculation (DFT) of the CAF-CA (form II) cocrystal has been done using monomer and dimer model. The dimer of CAF-CA cocrystal is able to incorporate all significant intra- and intermolecular interactions which we were not able to evaluate in its monomer model. The formed CAF-CA cocrystal was further analyzed and characterized by FT-IR, FT-Raman, PXRD and DSC techniques. The spectral features confirmed the presence of intermolecular hydrogen bonding interactions (C51 = O52∙∙∙H40-O39), (C38 = O41∙∙∙H89-O88) and (O26-H27∙∙∙N24-C22) in dimer model of cocrystal, resulting the red shift in C=O mode of CAF; C=O and O-H groups of CA along with elongation in bond lengths. The DSC pattern showed that melting point of CAF is higher than CA as well as CAF-CA cocrystal. Hence, confirmed the formed cocrystal. PXRD analysis predicted that almost perfect match has been seen in the simulated and observed diffraction pattern in cocrystal. AIM study suggested the nature of intermolecular hydrogen bond interaction (O88-H89∙∙∙O41) is partially covalent and possess maximum energy −12.4247 kcal mol^−1^. Further NBO analysis of dimer suggested that the interaction [n (1)N24 → σ*(O26-H27)] was responsible for stability of cocrystal with stabilization energy of 23.64 kcal mol^−1^. Also, intramolecular hydrogen bonding interactions [π(C51 = O52) → π*(N46-C51)]/[n (2)O39 → π*(C38 = O41)] within CAF/CA with stabilization energies 128.08/59.85 kcal mol^−1^, respectively provide extra stabilization to cocrystal. The HOMO-LUMO energy gap shows that cocrystal is more reactive (less stable) and softer than CAF (API). The calculated value of electrophilic charge transfer ECT is positive indicating that the charge flows from CA to CAF in cocrystal. The local reactivity descriptors suggested that in dimer model O90 and H10 atoms were regions susceptible for nucleophilic and electrophilic attack, respectively. MESP map displayed that nucleophilic/electropilic reactivity of groups involved in intermolecular hydrogen bonding were neutralized in dimer model. This map also represents that V_max_/V_min_ is for H85/O7 atoms, respectively representing them as electron deficient (blue region)/rich center (red region). The calculated MR value for dimer model (105.70 e. s.u) was within set range of Lipinski’s rule and hence it may be used as orally active form of drug. These results suggest that structural characterization, reactivity and hydrogen bonding interactions of CAF-CA cocrystal could be relevant in the field of pharmaceutical chemistry as well as industry.

## Data Availability

The original contributions presented in the study are included in the article/Supplementary Material, further inquiries can be directed to the corresponding authors.
