# Supplementary material for: Molecular Structure, Spectral Investigations, Hydrogen Bonding Interactions and Reactivity-Property Relationship of Caffeine-Citric Acid Cocrystal by Experimental and DFT Approach
Source: Front Chem. 2021 Jul 26;9:708538. doi: 10.3389/fchem.2021.708538 (PMC8350318; doi:10.3389/fchem.2021.708538)
Supplement: Supplementary file 1 [file DataSheet1.docx]

Molecular structure, spectral investigations, hydrogen bonding

interactions and reactivity-property relationship of caffeine-citric acid cocrystal by experimental and DFT approach

Priya Verma^1^, Anubha Srivastava^1^, Karnica Srivastava^2^, Poonam Tandon^1*^, Manishkumar R. Shimpi^3,4*^

Supplementary Material

# Supplementary Figures and Tables

The simulated and recorded PXRD pattern of caffeine-citric acid (CAF-CA) cocrystal is given in Figure S1. The DSC plot of cocrystal is shown in Figure S2. The cocrystal belongs to monoclinic system with space group P2_1_/c and unit cell parameters a= 13.7783(8) Å, b= 12.3149(8) Å, c=9.6587 (6) Å, β=92.854(4) Å, the crystal structure is shown in Figure S3. The optimized ground state structure of CAF, CA and monomer model of cocrystal are shown in Figures S4, S5 and S6, respectively. Experimental and calculated IR and Raman spectra of CAF and CA are shown in Figures S7, S8, S9 and S10, respectively. The molecular graph of a monomer model of cocrystal using AIM program is given in Figure S11. HOMO and LUMO plots of CAF, CA and monomer model of cocrystal with their energy gap are shown in Figures S11, S12, S13 and S14, respectively. The molecular electrostatic potential (MESP) surface of CAF, CA and monomer model of cocrystal are given in Figures S15, S16 and S17, respectively.

The experimental and calculated geometric parameters of CAF, monomer and dimer models of cocrystal are given in Table S1. Theoretical and experimental vibrational wavenumbers of CAF and CA are listed in Tables S2 and S3, respectively. The experimental and theoretical wavenumbers of the CAF-CA cocrystal for monomer and dimer models are given in Table S4. Geometrical and topological parameters for the intra- and intermolecular hydrogen bonds and the geometrical parameters for the existence of hydrogen bond interactions for monomer model of cocrystal are given in Tables S5 and S6, respectively. Geometrical parameters for the existence of hydrogen bond interactions for dimer model of cocrystal are given in Table S7. Geometrical parameter (bond length) and topological parameters for bonds of interacting atoms of intra- and intermolecular interactions of dimer model of cocrystal is given in Table S8. Second-order perturbation theory analyses of the Fock Matrix, in the NBO basis for intra- and intermolecular interactions in monomer and dimer models of cocrystal are given in Tables S9 and S10, respectively. Reactivity descriptors as Fukui functions ($f_{k}^{+}{, f}_{k}^{-}$), local softness ($s_{k}^{+}{, s}_{k}^{-})$, local electrophilicity indices ($\omega_{k}^{+}{, \omega}_{k}^{-})$ for monomer and dimer models of CAF-CA cocrystal using Hirshfeld atomic charges are given in Tables S11 and S12, respectively.

## Supplementary Figures


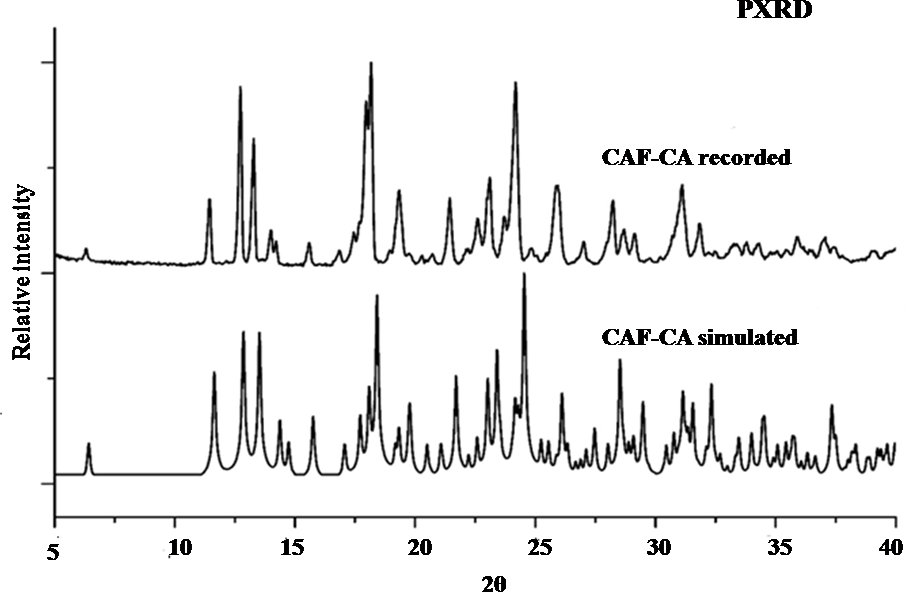


**Figure S1.** PXRD pattern of bulk CAF-CA cocrystal and the simulated PXRD pattern generated from known crystal structure (θ is in degree).

**
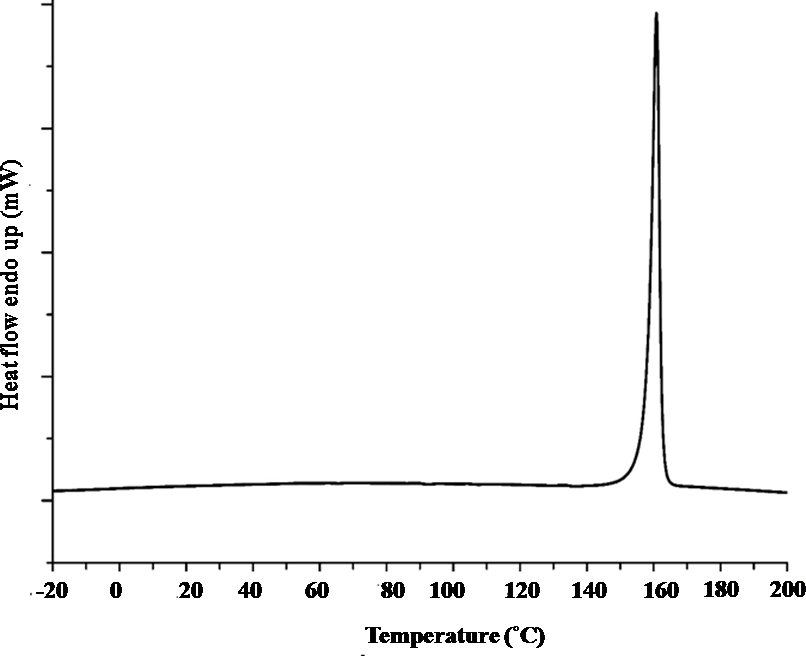
**

**Figure S2.** DSC thermogram on CAF-CA cocrystal.


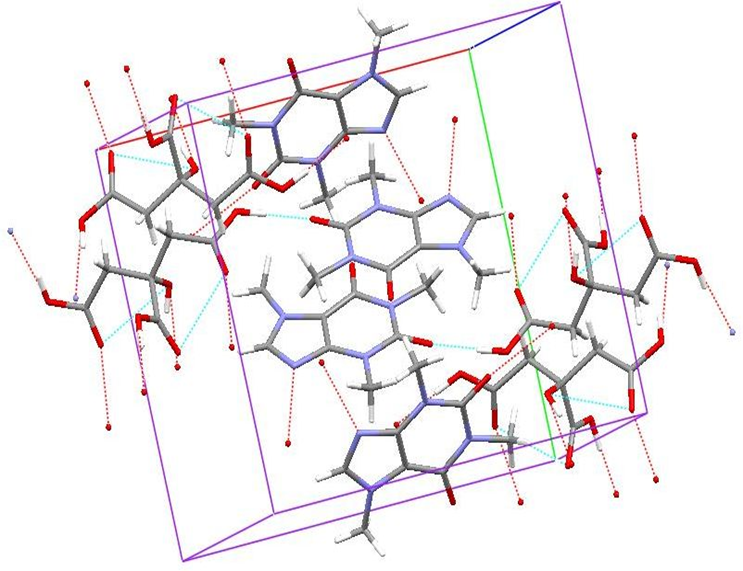


**Figure S3.** Interactions between CAF and CA molecules that are held together through hydrogen bonds in the crystal lattice of CAF-CA cocrystal (Cambridge Crystallographic Database (CSD) reference code is KIGKER01).


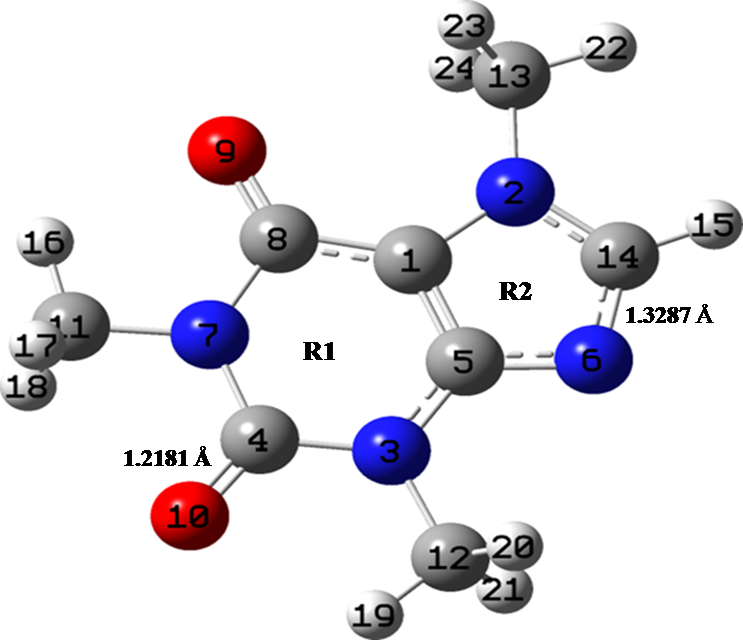


**Figure S4.** Optimized ground state structure of CAF and atomic numbering adopted in this study.


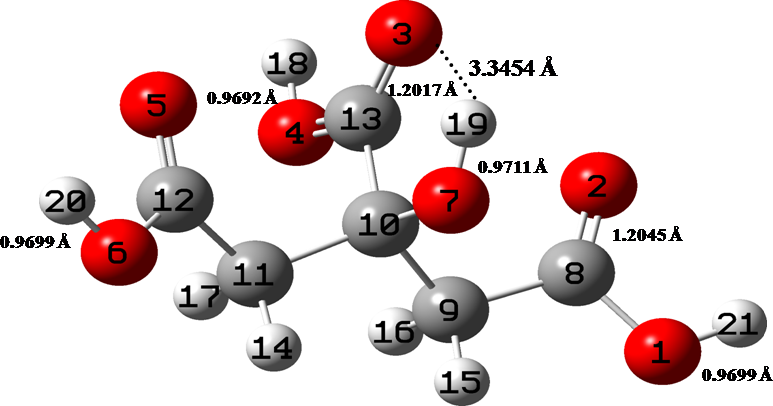


**Figure S5.** Optimized ground state structure of CA with atomic numbering adopted in this study.


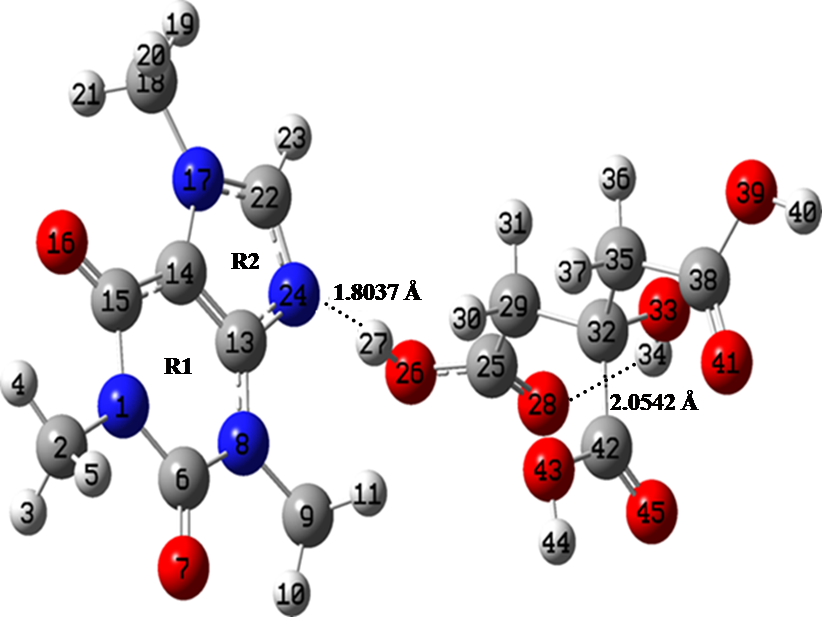


**Figure S6.** Optimized structure for monomer model of cocrystal with the atomic numbering scheme adopted in the present study.





**Figure S7 .**Experimental and calculated IR absorbance spectra of CAF in the region 400-1100 cm^−1^, 1100-1799 cm^−1^ and 2800-3150 cm^−1^.





**Figure S8.** Experimental and calculated Raman scattering spectra of CAF in the region 200-1000 cm^−1^, 1001-1770 cm^−1^ and 2890-3145 cm^−1^.





**Figure S9.** Experimental and calculated IR absorbance spectra of CA in the region 400-840 cm^-1^, 840-1490 cm^-1^ and 2850-3620 cm^-1^.





**Figure S10.** Experimental and calculated Raman scattering spectra of CA in the region 100-990 cm^-1^, 990-1860 cm^-1^ and 2900-3650 cm^-1^.


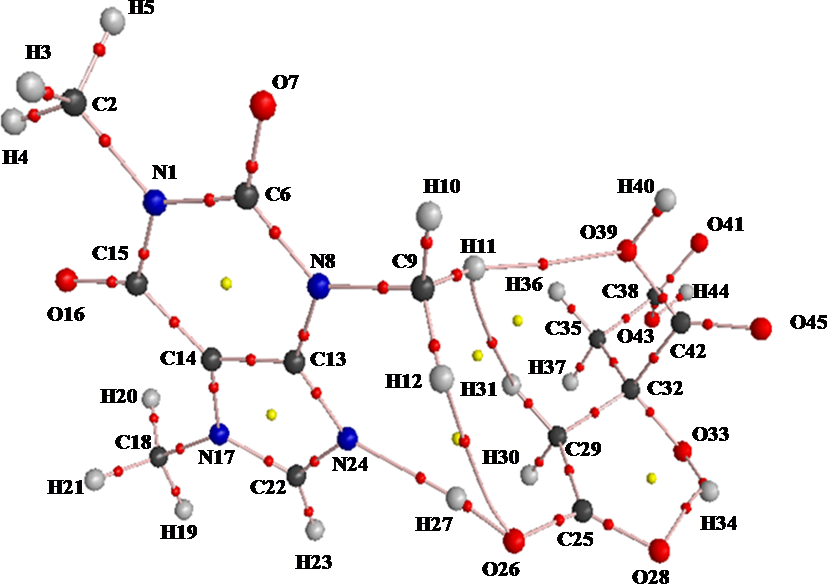


**Figure S11.** Molecular graph of monomer model of cocrystal: BCPs (small red spheres), ring critical points (small yellow sphere), bond paths (pink lines).


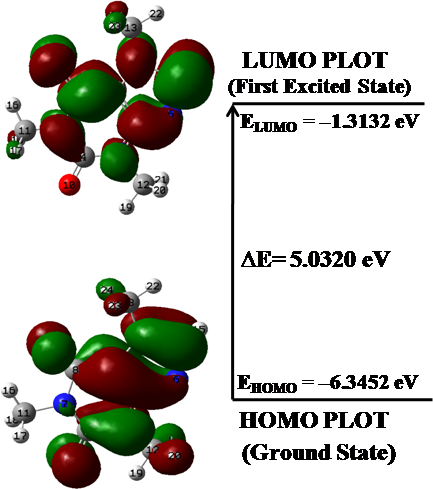


**Figure S12.** HOMO-LUMO plot showing energy gap (ΔE) of CAF with orbital involved in electronic transitions.


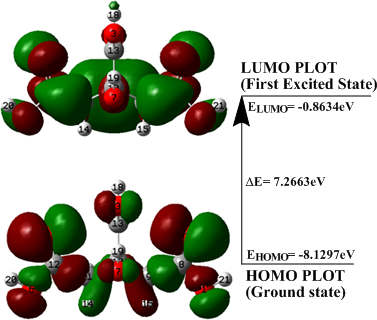


**Figure S13.** HOMO-LUMO plot showing energy gap (ΔE) of CA with orbital involved in electronic transitions.


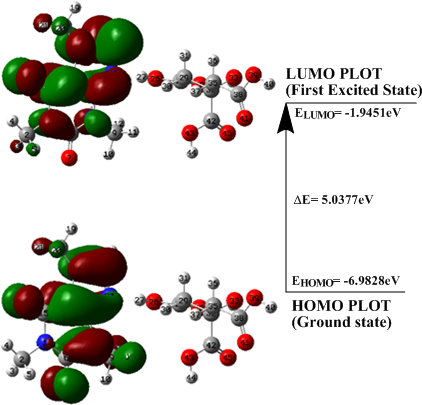


**Figure S14.** HOMO-LUMO plot showing energy gap (ΔE) of monomer model of cocrystal with orbital involved in electronic transitions.


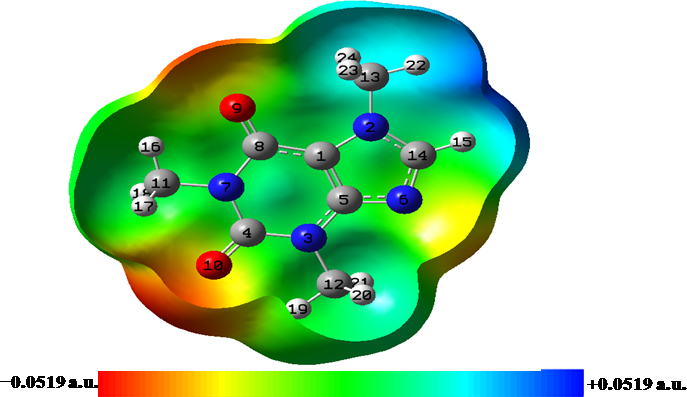


**Figure S15.** Molecular electrostatic potential (MESP) formed by mapping of total density over electrostatic potential in the gas phase for CAF.


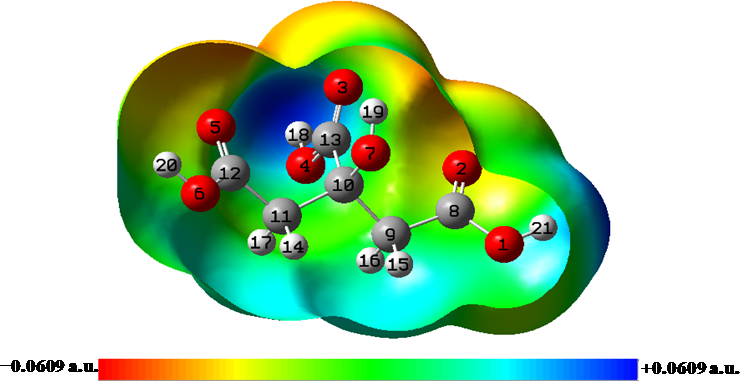


**Figure S16.** Molecular electrostatic potential (MESP) formed by mapping of total density over electrostatic potential in the gas phase for CA.


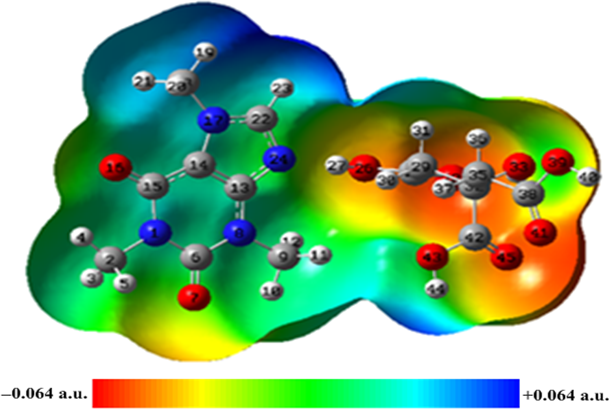


**Figure S17.** Molecular electrostatic potential (MESP) formed by mapping of total density over electrostatic potential in the gas phase for monomer model of cocrystal.

## Supplementary Tables

**Table S1.** The experimental parameters of CAF-CA cocrystal and calculated geometrical parameters of monomer and dimer models of cocrystal using DFT/6-311++G(d,p), bond lengths in angstroms (Å), bond angles and dihedral angles in degrees (˚).

| **Geometrical**  **parameters** | **Experimental** | **Optimized parameters** | **Experimental** | | | | **Computed optimized parameters** | | | | |  |
| --- | --- | --- | --- | --- | --- | --- | --- | --- | --- | --- | --- | --- |
|  | **CAF** | **CAF** | **CAF-CA**  **cocrystal** | | | | **monomer dimer**  **model model** | | | | |  |
|  |  | **Bond length (Å)** | | | | | | | | | |  |
| R(N1-C2) | 1.4658 | 1.4689 | 1.4777 | | | | 1.4705 | | | 1.4696 | |  |
| R(N1-C6) | 1.3874 | 1.4084 | 1.3952 | | | | 1.4080 | | | 1.4064 | |  |
| R(N1-C15) | 1.4210 | 1.4180 | 1.4141 | | | | 1.4140 | | | 1.4177 | |  |
| R(C2-H3) | 0.9999 | 1.0861 | 0.9799 | | | | 1.0899 | | | 1.0901 | |  |
| R(C2-H4) | 1.0001 | 1.0902 | 0.9794 | | | | 1.0860 | | | 1.0860 | |  |
| R(C2-H5) | 1.0000 | 1.0903 | 0.9810 | | | | 1.0900 | | | 1.0901 | |  |
| R(C6=O7) | 1.2226 | 1.2181 | 1.2397 | | | | 1.2152 | | | 1.2170 | |  |
| R(C6-N8) | 1.3715 | 1.3931 | 1.3618 | | | | 1.3984 | | | 1.3966 | |  |
| R(N8-C9) | 1.4666 | 1.4641 | 1.4733 | | | | 1.4666 | | | 1.4652 | |  |
| R(N8-C13) | 1.3712 | 1.3733 | 1.3804 | | | | 1.3701 | | | 1.3718 | |  |
| R(C9-H10) | 0.9998 | 1.0861 | 0.9811 | | | | 1.0861 | | | 1.0860 | |  |
| R(C9-H11) | 1.0000 | 1.0911 | 0.9805 | | | | 1.0923 | | | 1.0904 | |  |
| R(C9-H12) | 1.0000 | 1.0912 | 0.9800 | | | | 1.0902 | | | 1.0920 | |  |
| R(C13=C14) | 1.3652 | 1.3798 | 1.3661 | | | | 1.3780 | | | 1.3787 | |  |
| R(C13-N24) | 1.3544 | 1.3572 | 1.3656 | | | | 1.3635 | | | 1.3616 | |  |
| R(C14-C15) | 1.4126 | 1.4313 | 1.4177 | | | | 1.4348 | | | 1.4332 | |  |
| R(C14-N17) | 1.3858 | 1.3870 | 1.3898 | | | | 1.3868 | | | 1.3874 | |  |
| R(C15=O16) | 1.2201 | 1.2237 | 1.2295 | | | | 1.2218 | | | 1.2220 | |  |
| R(N17-C18) | 1.4618 | 1.4594 | 1.4757 | | | | 1.4624 | | | 1.4640 | |  |
| R(N17-C22) | 1.3372 | 1.3550 | 1.3517 | | | | 1.3471 | | | 1.3457 | |  |
| R(C18-H19) | 0.9999 | 1.0898 | 0.9790 | | | | 1.0894 | | | 1.0889 | |  |
| R(C18-H20) | 1.0001 | 1.0898 | 0.9802 | | | | 1.0893 | | | 1.0900 | |  |
| R(C18-H21) | 1.0001 | 1.0898 | 0.9807 | | | | 1.0892 | | | 1.0891 | |  |
| R(C22-H23) | 1.0002 | 1.0798 | 0.9501 | | | | 1.0793 | | | 1.0801 | |  |
| R(C22-N24) | 1.3281 | 1.3287 | 1.3416 | | | | 1.3346 | | | 1.3388 | |  |
| R(N24-H27) | - | - | 1.8275 | | | | 1.8037 | | | 1.8125 | |  |
| R(C25-O26) | - | - | 1.3330 | | | | 1.3357 | | | 1.3314 | |  |
| R(C25=O28) | - | - | 1.2182 | | | | 1.2091 | | | 1.2126 | |  |
| R(C25-C29) | - | - | 1.4951 | | | | 1.5268 | | | 1.5236 | |  |
| R(O26-H27) | - | - | 0.8876 | | | | 0.9919 | | | 0.9945 | |  |
| R(C29-H30) | - | - | 0.9894 | | | | 1.0910 | | | 1.0876 | |  |
| R(C29-H31) | - | - | 0.9896 | | | | 1.0959 | | | 1.0947 | |  |
| R(C29-C32) | - | - | 1.5368 | | | | 1.5511 | | | 1.5504 | |  |
| R(C32-O33) | - | - | 1.4157 | | | | 1.4032 | | | 1.4038 | |  |
| R(C32-C35) | - | - | 1.5329 | | | | 1.5427 | | | 1.5518 | |  |
| R(C32-C42) | - | - | 1.5219 | | | | 1.5486 | | | 1.5518 | |  |
| R(O33-H34) | - | - | 0.7940 | | | | 0.9711 | | | 0.9713 | |  |
| R(C35-H36) | - | - | 0.9906 | | | | 1.0899 | | | 1.0905 | |  |
| R(C35-H37) | - | - | 0.9892 | | | | 1.0924 | | | 1.0907 | |  |
| R(C35-C38) | - | - | 1.5081 | | | | 1.5151 | | | 1.5126 | |  |
| R(C38-O39) | - | - | 1.3257 | | | | 1.3515 | | | 1.3148 | |  |
| R(C38=O41) | - | - | 1.2150 | | | | 1.2045 | | | 1.2256 | |  |
| R(O39-H40) | - | - | 0.9801 | | | | 0.9699 | | | 0.9929 | |  |
| R(C42-O43) | - | - | 1.3390 | | | | 1.3631 | | | 1.3466 | |  |
| R(C42=O45) | - | - | 1.2119 | | | | 1.1958 | | | 1.2010 | |  |
| R(O43-H44) | - | - | 0.9581 | | | | 0.9693 | | | 0.9812 | |  |
|  |  | **Bond angle (Å)** | | | | | | | | | | |
| A(C2-N1-C6) | 116.79183 | 115.05748 | | 116.1939 | | | | 115.0718 | | | 115.0981 | |
| A(C2-N1-C15) | 116.50956 | 117.87689 | | 117.7751 | | | | 117.9509 | | | 117.8725 | |
| A(C6-N1-C15) | 126.69795 | 127.06558 | | 126.0306 | | | | 126.9768 | | | 127.0288 | |
| A(N1-C2-H3) | 109.50203 | 107.46604 | | 109.4889 | | | | 109.8731 | | | 109.9626 | |
| A(N1-C2-H4) | 109.49034 | 109.96929 | | 109.5075 | | | | 107.5059 | | | 107.4570 | |
| A(N1-C2-H5) | 109.51481 | 110.00843 | | 109.4492 | | | | 109.9084 | | | 109.9422 | |
| A(H3-C2-H4) | 109.44019 | 110.49976 | | 109.4725 | | | | 110.4961 | | | 110.4774 | |
| A(H3-C2-H5) | 109.43503 | 110.45646 | | 109.4493 | | | | 108.5617 | | | 108.4954 | |
| A(H4-C2-H5) | 109.44487 | 108.44179 | | 109.4598 | | | | 110.4924 | | | 110.5053 | |
| A(N1-C6=O7) | 121.64656 | 120.80217 | | 121.0683 | | | | 120.9404 | | | 120.9547 | |
| A(N1-C6-N8) | 117.36637 | 116.83449 | | 117.9921 | | | | 116.9928 | | | 116.9213 | |
| A(O7=C6-N8) | 120.98615 | 122.36335 | | 120.9396 | | | | 122.0667 | | | 122.1239 | |
| A(C6-N8-C9) | 119.43927 | 120.06538 | | 119.1959 | | | | 119.4845 | | | 119.6436 | |
| A(C6-N8-C13) | 119.3533 | 119.59965 | | 119.5592 | | | | 119.3489 | | | 119.4214 | |
| A(C9-N8-C13) | 121.20682 | 120.33483 | | 121.2436 | | | | 121.1634 | | | 120.9325 | |
| A(N8-C9-H10) | 109.51764 | 107.67376 | | 109.4235 | | | | 107.5436 | | | 107.6228 | |
| A(N8-C9-H11) | 109.49909 | 109.92615 | | 109.5007 | | | | 110.4489 | | | 110.0073 | |
| A(N8-C9-H12) | 109.50199 | 109.96263 | | 109.5404 | | | | 109.852 | | | 110.3905 | |
| A(H10-C9-H11) | 109.43171 | 110.2604 | | 109.3611 | | | | 109.4773 | | | 110.1295 | |
| A(H10-C9-H12) | 109.43869 | 110.23363 | | 109.4483 | | | | 110.0254 | | | 109.5923 | |
| A(H11-C9-H12) | 109.43814 | 108.77848 | | 109.5531 | | | | 109.4709 | | | 109.0859 | |
| A(N8-C13=C14) | 122.04496 | 121.94811 | | 121.2653 | | | | 122.0885 | | | 122.1352 | |
| A(N8-C13-N24) | 125.84567 | 126.32362 | | 126.6767 | | | | 127.1002 | | | 126.9314 | |
| A(C14=C13-N24) | 112.10608 | 111.72826 | | 112.0565 | | | | 110.8109 | | | 110.9331 | |
| A(C13=C14-C15) | 123.31041 | 123.37942 | | 123.5907 | | | | 123.3863 | | | 123.2654 | |
| A(C13=C14-N17) | 104.84861 | 105.14031 | | 105.2894 | | | | 105.6289 | | | 105.4757 | |
| A(C15-C14-N17) | 131.83651 | 131.48023 | | 131.1041 | | | | 130.9845 | | | 131.2589 | |
| A(N1-C15-C14) | 111.17029 | 111.17265 | | 111.5077 | | | | 111.2019 | | | 111.2250 | |
| A(N1-C15=O16) | 120.96367 | 122.42835 | | 121.2280 | | | | 122.8213 | | | 122.4566 | |
| A(C14-C15=O16) | 127.86244 | 126.3990 | | 127.2572 | | | | 125.9767 | | | 126.3184 | |
| A(C14-N17-C18) | 127.87566 | 127.02138 | | 127.1520 | | | | 126.8434 | | | 127.2876 | |
| A(C14-N17-C22) | 105.99683 | 105.6938 | | 106.1306 | | | | 106.1056 | | | 106.3377 | |
| A(C18-N17-C22) | 126.12734 | 127.28482 | | 126.6956 | | | | 127.0499 | | | 126.3738 | |
| A(N17-C18-H19) | 109.52709 | 108.28307 | | 109.4854 | | | | 108.3129 | | | 108.0783 | |
| A(N17-C18-H20) | 109.50238 | 110.2475 | | 109.4340 | | | | 110.0150 | | | 109.9345 | |
| A(N17-C18-H21) | 109.50055 | 110.24774 | | 109.4210 | | | | 109.9668 | | | 109.7272 | |
| A(H19-C18-H20) | 109.4362 | 109.73453 | | 109.4980 | | | | 109.9358 | | | 110.4051 | |
| A(H19-C18-H21) | 109.43083 | 109.73206 | | 109.5346 | | | | 109.8994 | | | 110.1050 | |
| A(H20-C18-H21) | 109.43019 | 108.58937 | | 109.4542 | | | | 108.7052 | | | 108.5857 | |
| A(N17-C22-H23) | 123.14282 | 121.74252 | | 123.4249 | | | | 122.3469 | | | 122.5877 | |
| A(N17-C22-N24) | 113.70532 | 113.6096 | | 113.0533 | | | | 113.052 | | | 112.8247 | |
| A(H23-C22-N24) | 123.15186 | 124.64788 | | 123.5219 | | | | 124.60 | | | 104.4273 | |
| A(C13-N24-C22) | 103.3428 | 103.82803 | | 103.4700 | | | | 104.4017 | | | 104.4273 | |
| A(C13-N24-H27) | - | - | | 127.7812 | | | | 133.3334 | | | 132.7865 | |
| A(C22-N24-H27) | - | - | | 125.7589 | | | | 121.3684 | | | 122.6551 | |
| A(O26-C25=O28) | - | - | | 118.9748 | | | | 120.8996 | | | 121.4245 | |
| A(O26-C25-C29) | - | - | | 117.3210 | | | | 116.2939 | | | 117.3008 | |
| A(O28=C25-C29) | - | - | | 123.6929 | | | | 122.7758 | | | 121.2638 | |
| A(C25-O26-H27) | - | - | | 111.2353 | | | | 113.8225 | | | 113.1189 | |
| A(C25-C29-H30) | - | - | | 108.9628 | | | | 110.4539 | | | 111.3109 | |
| A(C25-C29-H31) | - | - | | 108.9660 | | | | 106.7704 | | | 107.0980 | |
| A(C25-C29-C32) | - | - | | 112.9256 | | | | 113.7544 | | | 112.2095 | |
| A(H30-C29-H31) | - | - | | 107.7847 | | | | 107.4436 | | | 107.9490 | |
| A(H30-C29-C32) | - | - | | 107.7847 | | | | 110.7356 | | | 111.0322 | |
| A(H31-C29-C32) | - | - | | 109.0558 | | | | 107.3618 | | | 106.9708 | |
| A(C29-C32-O33) | - | - | | 111.6033 | | | | 111.5174 | | | 111.3411 | |
| A(C29-C32-C35) | - | - | | 108.4108 | | | | 108.3382 | | | 107.7116 | |
| A(C29-C32-C42) | - | - | | 110.4845 | | | | 109.4666 | | | 110.5130 | |
| A(O33-C32-C35) | - | - | | 105.1535 | | | | 106.3148 | | | 107.3176 | |
| A(O33-C32-C42) | - | - | | 110.0598 | | | | 110.1636 | | | 110.0992 | |
| A(C35-C32-C42) | - | - | | 111.0066 | | | | 110.9989 | | | 109.7681 | |
| A(C32-O33-H34) | - | - | | 109.8929 | | | | 107.2397 | | | 107.0114 | |
| A(C32-C35-H36) | - | - | | 108.9515 | | | | 108.1763 | | | 107.2811 | |
| A(C32-C35-H37) | - | - | | 109.0028 | | | | 109.6875 | | | 108.9843 | |
| A(C32-C35-C38) | - | - | | 113.1050 | | | | 112.7563 | | | 113.8275 | |
| A(H36-C35-H37) | - | - | | 107.7627 | | | | 109.1554 | | | 109.8383 | |
| A(H36-C35-C38) | - | - | | 108.9132 | | | | 109.763 | | | 109.3567 | |
| A(H37-C35-C38) | - | - | | 108.9690 | | | | 107.2575 | | | 107.5267 | |
| A(C35-C38-O39) | - | - | | 113.9683 | | | | 112.0894 | | | 113.8367 | |
| A(C35-C38=O41) | - | - | | 123.8154 | | | | 124.8784 | | | 123.0946 | |
| A(O39-C38=O41) | - | - | | 122.2059 | | | | 123.0028 | | | 123.0650 | |
| A(C38-O39-H40) | - | - | | 117.4542 | | | | 107.1701 | | | 111.2449 | |
| A(C32-C42-O43) | - | - | | 111.9225 | | | | 111.16 | | | 111.0164 | |
| A(C32-C42=O45) | - | - | | 124.4609 | | | | 125.496 | | | 124.7711 | |
| A(O43-C42=O45) | - | - | | 123.6129 | | | | 123.2991 | | | 124.2108 | |
| A(C42-O43-H44) | - | - | | 105.4772 | | | | 106.7573 | | | 110.3680 | |
| L(24,27,26,9,-1) | - | - | | - | | | | - | | | - | |
| L(24,27,26,9,-2) | - | - | | - | | | | - | | | - | |
|  |  | **Dihedral angle (Å)** | | | | | | | | | | |
| D(C6-N1-C2-H3) | 179.99939 | 179.6453 | | | −58.1157 | −59.6852 | | | −59.8056 | | | |
| D(C6-N1-C2-H4) | 60.00681 | 59.3175 | | | −178.1503 | −179.977 | | | 179.9028 | | | |
| D(C6-N1-C2-H5) | −59.99935 | −60.0567 | | | 61.8549 | 59.7149 | | | 59.5893 | | | |
| D(C15-N1-C2-H3) | −0.2746 | −0.4311 | | | 121.6646 | 120.5678 | | | 119.9339 | | | |
| D(C15-N1-C2-H4) | −120.26718 | −120.7589 | | | 1.6299 | 0.2758 | | | −0.3576 | | | |
| D(C15-N1-C2-H5) | 119.72667 | 119.8669 | | | −118.3649 | −120.032 | | | −120.6711 | | | |
| D(C2-N1-C6=O7) | 2.2031 | 0.0461 | | | 1.7844 | −0.2027 | | | 0.0903 | | | |
| D(C2-N1-C6-N8) | −177.45145 | −179.9595 | | | −178.3259 | 179.6541 | | | −179.7764 | | | |
| D(C15-N1-C6=O7) | −177.49111 | −179.8693 | | | −177.9751 | 179.5175 | | | −179.6213 | | | |
| D(C15-N1-C6-N8) | 2.85434 | 0.1251 | | | 1.9146 | −0.6257 | | | 0.5121 | | | |
| D(C2-N1-C15-C14) | 178.56721 | −179.9737 | | | 178.1393 | 179.9282 | | | −179.8684 | | | |
| D(C2-N1-C15=O16) | −0.79398 | 0.03803 | | | −2.7584 | −0.0518 | | | 0.1526 | | | |
| D(C6-N1-C15-C14) | −1.73782 | −0.0604 | | | −2.1046 | 0.2151 | | | −0.1639 | | | |
| D(C6-N1-C15=O16) | 178.90099 | 179.9513 | | | 176.9977 | −179.765 | | | 179.8572 | | | |
| D(N1-C6-N8-C9) | 178.62694 | −179.9655 | | | −179.3525 | −179.825 | | | 179.9148 | | | |
| D(N1-C6-N8-C13) | −1.65354 | −0.1032 | | | 0.2423 | 0.8287 | | | −0.6552 | | | |
| D(O7=C6-N8-C9) | −1.03002 | 0.02885 | | | 0.5374 | 0.0299 | | | 0.0498 | | | |
| D(O7=C6-N8-C13) | 178.68949 | 179.8911 | | | −179.8679 | −179.316 | | | 179.4798 | | | |
| D(C6-N8-C9-H10) | −0.28107 | −0.40672 | | | 23.6332 | 8.8968 | | | −6.5799 | | | |
| D(C6-N8-C9-H11) | −120.2782 | −120.5454 | | | −96.2209 | −110.516 | | | −126.5763 | | | |
| D(C6-N8-C9-H12) | 119.72639 | 119.7206 | | | 143.6181 | 128.6306 | | | 112.9865 | | | |
| D(C13-N8-C9-H10) | −179.99524 | 179.7320 | | | −155.9546 | −171.769 | | | 173.9988 | | | |
| D(C13-N8-C9-H11) | 60.00763 | 59.5934 | | | 84.1913 | 68.8182 | | | 54.0025 | | | |
| D(C13-N8-C9-H12) | −59.98778 | −60.1406 | | | −35.9696 | −52.0353 | | | −66.4347 | | | |
| D(C6-N8-C13=C14) | −0.36241 | 0.0301 | | | −1.9563 | −0.6943 | | | 0.5074 | | | |
| D(C6-N8-C13-N24) | −179.64930 | −179.9850 | | | 178.5184 | 179.5629 | | | −179.7022 | | | |
| D(C9-N8-C13=C14) | 179.35200 | 179.8920 | | | 177.6300 | 179.9707 | | | 179.9299 | | | |
| D(C9-N8-C13-N24) | 0.06511 | −0.1231 | | | −1.8954 | 0.228 | | | −0.2797 | | | |
| D(N8-C13=C14-C15) | 1.51138 | 0.0378 | | | 1.6931 | 0.2765 | | | −0.1478 | | | |
| D(N8-C13=C14-N17) | −179.17598 | 179.9713 | | | −179.6135 | −179.561 | | | 179.8003 | | | |
| D(N24-C13=C14-C15) | −179.11252 | −179.9491 | | | −178.7176 | −179.943 | | | −179.9684 | | | |
| D(N24-C13=C14-N17) | 0.20012 | −0.0156 | | | −0.0242 | 0.2194 | | | −0.0203 | | | |
| D(N8-C13-N24-C22) | 179.18927 | −179.9803 | | | 179.4949 | 179.4704 | | | −179.5657 | | | |
| D(N8-C13-N24-H27) | - | - | | | −19.4262 | 10.6193 | | | −3.7840 | | | |
| D(C14=C13-N24-C22) | −0.15832 | 0.0059 | | | −0.0674 | −0.2964 | | | 0.2442 | | | |
| D(C14=C13-N24-H27) | - | - | | | 161.0116 | −169.148 | | | 176.0260 | | | |
| D(C13=C14-C15-N1) | −0.48411 | −0.0234 | | | 0.2729 | −0.0224 | | | −0.0318 | | | |
| D(C13=C14-C15=O16) | 178.82206 | 179.9643 | | | −178.7626 | 179.9569 | | | 179.9462 | | | |
| D(N17-C14-C15-N1) | −179.59233 | −179.9377 | | | −178.0544 | 179.7705 | | | −179.9652 | | | |
| D(N17-C14-C15=O16) | −0.28617 | 0.0499 | | | 2.9101 | −0.2503 | | | 0.0127 | | | |
| D(C13=C14-N17-C18) | 179.98728 | 179.9848 | | | −178.2756 | 179.5877 | | | 179.4503 | | | |
| D(C13=C14-N17-C22) | −0.15611 | 0.0185 | | | 0.1058 | −0.0505 | | | −0.2130 | | | |
| D(C15-C14-N17-C18) | −0.78372 | −0.0893 | | | 0.2799 | −0.2326 | | | −0.6074 | | | |
| D(C15-C14-N17-C22) | 179.07289 | 179.9443 | | | 178.6613 | −179.871 | | | 179.7292 | | | |
| D(C14-N17-C18-H19) | 179.99095 | 179.9954 | | | −176.2299 | −179.415 | | | −175.8244 | | | |
| D(C14-N17-C18-H20) | 59.98049 | 59.9353 | | | 63.7514 | 60.3992 | | | 63.6099 | | | |
| D(C14-N17-C18-H21) | −60.00631 | −59.9474 | | | −56.1744 | −59.3025 | | | −55.7455 | | | |
| D(C22-N17-C18-H19) | 0.16159 | −0.0453 | | | 5.7094 | 0.1494 | | | 3.7744 | | | |
| D(C22-N17-C18-H20) | −119.84886 | −120.1054 | | | −114.3094 | −120.036 | | | −116.7913 | | | |
| D(C22-N17-C18−H21) | 120.16434 | 120.0119 | | | 125.7649 | 120.262 | | | 123.8533 | | | |
| D(C14−N17−C22−H23) | -179.94385 | 179.9850 | | | 179.8001 | 179.4957 | | | -179.4501 | | | |
| D(C14-N17-C22-N24) | 0.06742 | -0.0164 | | | −0.1592 | −0.1399 | | | 0.3898 | | | |
| D(C18-N17-C22-H23) | −0.08397 | 0.0188 | | | −1.8088 | −0.1415 | | | 0.8825 | | | |
| D(C18-N17-C22-N24) | 179.9273 | −179.9827 | | | 178.2319 | −179.777 | | | −179.2775 | | | |
| D(N17-C22-N24-C13) | 0.05235 | 0.0068 | | | 0.1404 | 0.2686 | | | −0.3938 | | | |
| D(N17-C22-N24-H27) | - | - | | | −161.4492 | 170.7877 | | | −176.7177 | | | |
| D(H23-C22-N24-C13) | −179.93638 | −179.9947 | | | −179.8189 | −179.357 | | | 179.4424 | | | |
| D(H23-C22-N24-H27) | - | - | | | 18.5916 | −8.8383 | | | 3.1185 | | | |
| D(C13-N24-O26-C25) | - | - | | | −145.4268 | −104.983 | | | 143.0322 | | | |
| D(C22-N24-O26-C25) | - | - | | | 12.1601 | 92.7281 | | | −45.4592 | | | |
| D(O28=C25-O26-H27) | - | - | | | 166.1585 | 176.0024 | | | 170.3817 | | | |
| D(C29-C25-O26-H27) | - | - | | | −15.0219 | −5.9601 | | | −10.8087 | | | |
| D(O26-C25-C29-H30) | - | - | | | 63.5398 | 35.2451 | | | 14.2176 | | | |
| D(O26-C25-C29-H31) | - | - | | | −53.8211 | −81.2771 | | | −103.5626 | | | |
| D(O26-C25-C29-C32) | - | - | | | −175.1661 | 160.4849 | | | 139.3439 | | | |
| D(O28=C25-C29-H30) | - | - | | | −117.7015 | −146.758 | | | −166.9708 | | | |
| D(O28=C25-C29-H31) | - | - | | | 124.9377 | 96.72 | | | 75.2490 | | | |
| D(O28=C25-C29-C32) | - | - | | | 3.5927 | −21.518 | | | −41.8445 | | | |
| D(C25-C29-C32-O33) | - | - | | | 63.1074 | 58.7515 | | | 67.9300 | | | |
| D(C25-C29-C32-C35) | - | - | | | 178.4566 | 175.418 | | | −174.6659 | | | |
| D(C25-C29-C32-C42) | - | - | | | −59.6955 | −63.4126 | | | −54.7607 | | | |
| D(H30-C29-C32-O33) | - | - | | | −175.6295 | −176.159 | | | −166.7907 | | | |
| D(H30-C29-C32-C35) | - | - | | | −60.2802 | −59.4923 | | | −49.3867 | | | |
| D(H30-C29-C32-C42) | - | - | | | 61.5677 | 61.677 | | | 70.5186 | | | |
| D(H31-C29-C32-O33) | - | - | | | −58.1868 | −59.1459 | | | −49.2395 | | | |
| D(H31-C29-C32-C35) | - | - | | | 57.1624 | 57.5206 | | | 68.1645 | | | |
| D(H31-C29-C32-C42) | - | - | | | 179.0104 | 178.6899 | | | −171.9303 | | | |
| D(C29-C32-O33-H34) | - | - | | | −82.9409 | −61.3311 | | | −55.2000 | | | |
| D(C35-C32-O33-H34) | - | - | | | 159.7255 | −179.222 | | | −172.8426 | | | |
| D(C42-C32-O33-H34) | - | - | | | 40.1042 | 60.4306 | | | 67.7283 | | | |
| D(C29-C32-C35-H36) | - | - | | | −63.2568 | −60.0507 | | | −61.7729 | | | |
| D(C29-C32-C35-H37) | - | - | | | 54.0920 | 58.9148 | | | 57.1008 | | | |
| D(C29-C32-C35-C38) | - | - | | | 175.4713 | 178.3718 | | | 177.1058 | | | |
| D(O33-C32-C35-H36) | - | - | | | 56.2262 | 59.9253 | | | 58.2103 | | | |
| D(O33-C32-C35-H37) | - | - | | | 173.5749 | 178.8909 | | | 177.0840 | | | |
| D(O33-C32-C35-C38) | - | - | | | −65.0458 | −61.6522 | | | −62.9110 | | | |
| D(C42-C32-C35-H36) | - | - | | | 175.2156 | 179.7314 | | | 177.8513 | | | |
| D(C42-C32-C35-H37) | - | - | | | −67.4356 | −61.303 | | | −63.2750 | | | |
| D(C42-C32-C35-C38) | - | - | | | 53.9437 | 58.1539 | | | 56.7300 | | | |
| D(C29-C32-C42-O43) | - | - | | | −50.5932 | −58.8542 | | | −42.5909 | | | |
| D(C29-C32-C42=O45) | - | - | | | 128.7298 | 118.7664 | | | 137.8671 | | | |
| D(O33-C32-C42-O43) | - | - | | | −174.2918 | 178.1748 | | | −166.0037 | | | |
| D(O33-C32-C42=O45) | - | - | | | 5.0312 | −4.2046 | | | 14.4544 | | | |
| D(C35-C32-C42-O43) | - | - | | | 69.7117 | 60.6912 | | | 76.0701 | | | |
| D(C35-C32-C42=O45) | - | - | | | −110.9654 | −121.688 | | | −103.4718 | | | |
| D(C32-C35-C38-O39) | - | - | | | 122.8827 | 124.8337 | | | 98.1317 | | | |
| D(C32-C35-C38=O41) | - | - | | | −55.9631 | −57.0928 | | | −82.5449 | | | |
| D(H36-C35-C38-O39) | - | - | | | 1.5892 | 4.1594 | | | −21.8247 | | | |
| D(H36-C35-C38=O41) | - | - | | | −177.2566 | −177.767 | | | 157.4986 | | | |
| D(H37-C35-C38-O39) | - | - | | | −115.7190 | −114.312 | | | −141.0443 | | | |
| D(H37-C35-C38=O41) | - | - | | | 65.4353 | 63.7615 | | | 38.2791 | | | |
| D(C35-C38-O39-H40) | - | - | | | 177.1890 | 179.8457 | | | −176.8134 | | | |
| D(O41=C38-O39-H40) | - | - | | | −3.9443 | 1.7302 | | | 3.8630 | | | |
| D(C32-C42-O43-H44) | - | - | | | 172.8774 | −176.235 | | | −176.8155 | | | |
| D(O45=C42-O43-H44) | - | - | | | −6.4523 | 6.0822 | | | 2.7295 | | | |

**Table S2.** Theoretical and experimental vibrational wavenumbers (cm^-1^) of CAF and their assignments using B3LYP/6-311++G(d,p).

| **Unscaled** | **Scaled** | **IR** | **Raman** | **Potential Energy Distribution (**$\boldsymbol{\geq}\boldsymbol{5}\boldsymbol{\%}$**)** |
| --- | --- | --- | --- | --- |
| 3243 | 3100 | 3111 | 3114 | R2[ν(C14H15)](99) |
| 3175 | 3038 | 3028 | 3039 | R1[ν_a_(C12H_3_)](96) |
| 3174 | 3038 |  |  | R1[ν_a_(C11H_3_)](96) |
| 3142 | 3009 | 3018 | 3009 | R2[ν_a_(C13H_3_)](100) |
| 3131 | 2999 |  |  | R2[ν(C13H24)](50)+R2[ν(C13H23)](50) |
| 3122 | 2990 | 2991 | 2996 | R1[ν(C11H17)](51)+R2[ν(C11H18)](49) |
| 3112 | 2981 | 2981 |  | R1[ν(C12H20)](50)+R2[ν(C12H21)](49) |
| 3061 | 2935 | 2954 | 2958 | R1[ν_s_(C11H_3_)](99) |
| 3059 | 2933 |  |  | R2[ν_s_(C13H_3_)](99) |
| 3053 | 2928 |  |  | R1[ν_s_(C12H_3_)](99) |
| 1754 | 1719 | 1696 | 1699 | R1[ν(C4=O10)](42)+R1[ν(C8=O9)](29)+R1[δ_tri_](8)+R1[ν(C1C8)](7) |
| 1713 | 1680 | 1656 | 1655 | R1[ν(C8=O9)](35)+[ν(C4=O10)](32)+R1[ν(C1C8)](10) |
| 1624 | 1595 | 1598 | 1601 | [ν(C1=C5)](46)+R1[ν(C5N3)](26)+R2[δ](8)+[ν(C8=O9)](5) |
| 1574 | 1548 | 1547 | 1555 | R2[ν(CN)](15)+R2[ν(C1N2)](14)+R1[ν(C5N3)](11)+R1[ν(C1C8)](8)+R1[δ_a_](7)+R1[δ_tri_](6)+ R2[δ_in_(C14H15)](6) |
| 1524 | 1499 |  | 1491 | R2[δ’_a_(C13H_3_)](28)+R2[ν(C14N2)](19)+R2[ρ(C13H_3_)](11)+R2[δ_sym_(C13H_3_)](10)+R2[δ_a_(C13H_3_)](9)+  R2[δ_in_(C14H15)](6)+R2[δ’](5) |
| 1509 | 1485 |  |  | R1[δ’_a_(C12H_3_)](41)+R1[δ_a_(C12H_3_)](12)+R1[ρ(C12H_3_)](10)+R2[δ’_a_(C13H_3_)](10)+R1[δ_sym_(C12H_3_)(5)] |
| 1507 | 1483 | 1484 | 1483 | R1[δ’_a_(C11H_3_)](55)+R1[δ_a_(C11H_3_)](17)+R1[ρ(C11H_3_)](13)+R1[δ_sym_(C11H_3_)](8) |
| 1497 | 1473 |  |  | R1[δ_a_(C12H_3_)](69)+R1[δ’_a_(C12H_3_)](21)+R1[ρ’(C12H_3_)](9) |
| 1493 | 1470 |  | 1469 | R1[δ_a_(C11H_3_)](68)+R1[δ’_a_(C11H_3_)](22)+R1[ρ’(C11H_3_)](9) |
| 1490 | 1467 |  |  | R2[δ’_a_(C13H_3_)](16)+R2[δ_sym_(C13H_3_)](10)+R1[δ’_a_(C12H_3_)](10)+ R2[δ’](9)+R2[ν(C14N2)](6)+ R1[δ_sym_(C12H_3_)](6)+R1[ν(C5N3)](5)+R2[δ_a_(C13H_3_)](5) |
| 1477 | 1455 | 1454 | 1458 | R2[δ_a_(C13H_3_)](66)+R2[δ’_a_(C13H_3_)](22)+R2[ρ’(C13H_3_)](11) |
| 1459 | 1437 |  |  | R1[δ_sym_(C11H_3_)(34)+R2[δ_sym_(C12H_3_)(17)]+R2[δ_sym_(C13H_3_)(8)+R2[ν(C14N6)](7) |
| 1449 | 1428 | 1428 | 1430 | R2[δ_sym_(C13H_3_)(55)+[ν(C13N2)](9)+R1[δ_sym_(C12H_3_)](9)+R2[ν(C14N2)](6)+R2[δ_in_(C14H15)](5) |
| 1438 | 1417 | 1400 | 1409 | R1[δ_sym_(CH_3_)](72) |
| 1407 | 1387 | 1372 | 1374 | R2[ν(C14N6)](15)+R2[ν(C1N2)(18)+R1[δ_sym_(C12H_3_)(15)]+R2[ν(C13N2)(8)+R1[ν(C5N3)](8)+ R2[ν(C5N6)](6) |
| 1381 | 1362 | 1358 | 1361 | R2[ν(C14N2)(20)+R2[ν(C14N6)](12)+R2[ν(C1N2)(12)+R2[ρ(C13H_3_)](7)+[δ_in_(C13N2)](6)+R2[δ’_a_(C13H_3_)](6)+ R2[ν(C5N6)](6) |
| 1353 | 1335 | 1325 | 1329 | R2[ν(C14N6)](13)+R2[ν(C5N6)](12)R1[ν(C8N7)](10)+[ν(C11N7)](10)+R1[ν(C4N3)](9)+R1[δ_in_(C4=O10)](7)+R1[δ_sym_(C11H_3_)(6)+ν(C4N7)(5)+[ν(C1=C5)](5) |
| 1305 | 1289 | 1284 | 1285 | R1[ν(C4N3)](18)+ν(C12N3)(17)+R2[ν(CN)(13)+R1[ν(C1C8)](12)+R1[ρ(C12H_3_)](5) |
| 1268 | 1253 |  | 1251 | R1[ρ(C11H_3_)(23)+R1[ν(C8N7)](15)+R1[ν(C4N7)](10)+R2[δ_in_(C14H15)(10)+[δ_in_(C11N7)](8)+ R1[δ’_a_(C11H_3_)(6)+R2[ν(C14N6)](5) |
| 1260 | 1246 | 1238 | 1241 | R1[ν(C4N7)(18)+R2[δ_in_(C14H15)](16)+R1[ρ(C12H_3_)](9)+[ν(C11N7)](9)+R2[ν(C5N6)](8)+R1[ρ(C11H_3_)](5) |
| 1227 | 1213 | 1216 | 1213 | [ν(C12N3)(16)+R1[δ_tri_](16)+[ν(C13N2)(16)+R2[δ’](12)+[ν(C11N7)](9) |
| 1213 | 1199 | 1186 | 1191 | R2[δ_in_(C14H15)](29)+R1[ρ(C12H_3_)](18)+R2[ν(C5N6)](18)+[ν(C13N2)](5) |
| 1154 | 1142 |  |  | R1[ρ’(C12H_3_)](61)+R1[ρ’(C11H_3_)](28)+R1[δ_a_(C12H_3_)](5) |
| 1152 | 1140 |  |  | R1[ρ’(C11H_3_)](60)+R1[ρ’(C12H_3_)](28) |
| 1147 | 1136 | 1131 | 1134 | R2[ρ’(C13H_3_)](87)+R1[δ_a_(C13H_3_)](8) |
| 1085 | 1076 | 1071 | 1071 | R2[ρ(C13H_3_)](53)+R2[ν(C14N2)](19)+R2[δ](7) |
| 1070 | 1061 |  |  | R1[ρ(C12H_3_)](21)+R1[ν(C4N3)](19)+[ν(C11N7)](13)+R1[ν(C5N3)](8)+R2[δ](6)+R2[ν(C1N2)](5) |
| 1034 | 1026 | 1024 | 1022 | R1[ν(C4N7)](22)+R1[ν(C8N7)](22)+R1[ρ(C11H_3_)](21)+[ν(C12N3)](8)+R2[δ](5) |
| 988 | 981 | 972 | 976 | [ν(C12N3)](26)+R2[δ](19)+[ν(C11N7)](14)+R1[ν(C8N7)](8)+R1[ρ(C11H_3_)](6) |
| 938 | 932 | 926 | 928 | [ν(C11N7)](19)+R1[δ_in_(C8=O9)](13)+R1[ρ(C12H_3_)](10)+R2[ν(C1N2)](9)+R1[ν(C4N3)](9)+R2[δ](6)+R1[ν(C1C8)](6) |
| 837 | 833 | 828 | 825 | R2[oop(C14H15)](82)+R2[τ](8)+R2[τ’](7) |
| 815 | 811 | 800 | 802 | R1[δ_in_(C4=O10)](22)+R2[δ](18)+R1[δ_in_(C8=O9)(13)+R1[δ_in_(C11N7)](8)+[ν(C12N3)](5) |
| 761 | 758 | 759 | 766 | R1[oop(C8=O9)(33)+R1[puck](26)+R2[τ’](14)+R1[τ_a_](10)+R2[τ](10) |
| 754 | 752 |  | 760 | R1[oop(C4=O10)(57)+R1[puck](25)+R1[τ_a_](7)+[oop(C11N7)](5) |
| 749 | 747 | 744 | 741 | [ν(C13N2)](21)+R1[δ_tri_](12)+R1[δ_in_(C4=O10)](10)+R2[δ’](10)+R2[δ](9)+R1[δ_in_(C12N3)](6)+  R1[δ_in_(C8=O9)](6) |
| 707 | 705 | 700 | 698 | R2[τ’](38)+R1[oop(C8=O9)](25)+R1[τ’_a_](13)+[τ(C5=C1)](10)+[oop(C11N7)](5) |
| 645 | 644 | 644 | 644 | R1[δ_tri_](41)+[ν(C13N2)](13)+R2[δ’](9) |
| 613 | 612 | 608 | 610 | R2[τ](58)+R2[τ’](18)+[oop(C13N2)](12) |
| 552 | 552 | 550 | 556 | R1[ν(C4N7)](12)+R1[ν(C8N7)](12)+[ν(C12N3)](11)+R1[ν(C1C8)](10)+R1[ν(C5N3)](8)+R2[ν(C1N2)](7)+  R1[ν(C4N3)](7)+[ν(C11N7)](7)+[δ_in_(C13N2)](7) |
| 483 | 483 | 480 | 484 | R1[δ_a_](45)+R2[δ’](10)+[δ_in_(C13N2)](8)+R1[δ’_a_](5)+R1[ν(C1C8)](5) |
| 444 | 445 | 444 | 444 | R1[δ’_a_](43)+R1[δ_a_](8)+R1[δ_in_(C4=O10)](8)+R1[ν(C4N7)](7) |
| 423 | 424 | 423 | 422 | R1[δ_in_(C4=O10)](39)+R1[δ’_a_](19)+[δ_in_(C13N2)](8)+R1[ν(C5N3)](6)+R2[δ’](6) |
| 395 | 396 | - | 391 | R1[δ_in_(C8=O9)](48)+[δ_in_(C13N2)](18)+[δ_in_(C11N7)](7)+R1[δ’_a_](5)+R1[ν(C1C8)](5) |
| 361 | 362 | - | 366 | [oop(C12N3)](26)+R1[puck](18)+R1[τ’_a_](17)]+R2[τ](14)+[oop(C13N2)](13)+R2[τ’](7) |
| 359 | 360 | - | 349 | [δ_in_(C11N7)](53)+R2[δ_in_(C13N2)](13)+R1[δ’_a_](6) |
| 301 | 302 | - | 297 | [δ_in_(C12N3)]( 65)+[δ_in_(C11N7)](11) |
| 270 | 271 | - | 276 | [oop(C11N7)](62)+R1[τ’_a_](13)+[τ(C5=C1)](9)+[oop(C12N3)](6)+R1[puck](5) |
| 214 | 215 | - | 227 | R1[τ_a_](32)+[τ(C5=C1)](23)+[oop(C13N2)](21)+[oop(C12N3)](19)] |
| 205 | 206 | - | 186 | [δ_in_(C13N2)](38)+R1[δ_a_](15)+R2[ν(C1N2)](7)+R1[ν(C1C8)](5) |
| 152 | 153 | - | 144 | R1[puck](48)+[τ(C5=C1)](34)+[oop(C11N7)](8) |
| 122 | 123 | - | 126 | [τ(C5=C1)](34)+[oop(C12N3)](22)+R1[τ_a_](20)+[oop(C11N7)](10)+[oop(C13N2)](5) |
| 111 | 112 | - | 106 | R1[τ_a_](28)+[τ(C11N7)](19)+[oop(C12N3)](15)+R1[τ’_a_](9)+[τ(C13N2)](9)+[τ(C5=C1)](7) |
| 87 | 88 | - | - | R1[τ’_a_](57)+R1[τ_a_](29)]+[τ(C13N2)](6) |
| 77 | 78 | - | - | [τ(C11N7)](31)+R1[puck](24)+R1[τ_a_](13)+[oop(C11N7)](10)+R1[τ’_a_](7)+R1[ρ’(C11H_3_)](5) |
| 57 | 57 | - | - | R2[τ(C13N2)](35)+[oop(C13N2)](23)+[τ(C5=C1)](18)+[R1(τ_a_)](10)+R1[puck](6) |
| 14 | 14 | - | - | [τ(C12N3)](32)+[oop(C12N3)](16)+R1[ρ’(C12H_3_)](15)+R1[puck](14)+R1[τ_a_](8)+R1[δ_a_(C12H_3_)](5) |

**Table S3.** Theoretical and experimental vibrational wavenumbers (cm^-1^) of CA and their assignments using B3LYP/6-311++G(d,p).

| **Unscaled** | **Scaled** | **IR** | **Raman** | **Potential Energy Distribution (**$\boldsymbol{\geq}\boldsymbol{5}\boldsymbol{\%}$**)** |
| --- | --- | --- | --- | --- |
| 3763 | 3565 | 3493 | 3496 | [ν(O4H18)](100) |
| 3750 | 3553 | 3286 | 3291 | [ν(O6H20)](61)+[ν(O1H21)](39) |
| 3750 | 3553 |  |  | [ν(O1H21)](61)+[ν(O6H20)](39) |
| 3698 | 3507 | 3224 |  | [ν(O7H19)](100) |
| 3120 | 2989 | 2992 | 2993 | [ν_a_(C9H_2_)] (50)+[ν_a_(C11H_2_)](49) |
| 3114 | 2983 | 2979 | 2979 | [ν_a_(C11H_2_)](51)+[ν_a_(C9H_2_)](49) |
| 3061 | 2935 |  | 2930 | [ν_s_(C11H_2_)](50)+[ν_s_(C9H_2_)](50) |
| 3054 | 2929 |  |  | [ν_s_(C9H_2_)](50)+[ν_s_(C11H_2_)](50) |
| 1833 | 1794 |  |  | [ν(C13=O3)](45)+[ν(C12=O5)](18)+[ν(C8=O2)](17) |
| 1813 | 1776 | 1744 | 1737 | [ν(C8=O2)](40)+[ν(C12=O5)](40) |
| 1810 | 1772 |  |  | [ν(C13=O3)](36)+[ν(C8=O2)](22)+[ν(C12=O5)](22) |
| 1472 | 1449 | 1439 | 1433 | [δ_sci_(C9H_2_)](41)+[δ_sci_(C11H_2_)](41) |
| 1453 | 1431 |  |  | [δ_sci_(C11H_2_)](45)+[δ_sci_(C9H_2_)](44) |
| 1442 | 1421 | 1427 | 1416 | [δ(C10H19O7)](20)+[ω(C11H_2_)](12)+[ω(C9H_2_)](12)+[ν(C10C13)(10)+[γ(C9H_2_)]  (5)+[γ(C11H_2_)](5)+[ν(C13O4)](5) |
| 1392 | 1373 | 1388 | 1389 | [δ(C10H19O7)](21)+[ω(C11H_2_)](11)+[ω(C9H_2_)](11)+[ν(CC)](10)+[δ_sci_(C11H_2_)]  (5) |
| 1386 | 1367 | 1357 | 1365 | [ω(C9H_2_)](18)+[ω(C11H_2_)](17)+[ν(CC)](16)+[ν(CO)](12)+[δ(C8H21O1)](5)+ [δ(C12H20O6)](5) |
| 1340 | 1322 | 1320 | 1316 | [δ(C13H18O4)](30)+[δ(C10H19O7)](26)+[ν(C13O4)](16)+[δ_sym_(O4C13)](10) |
| 1311 | 1294 | 1292 | 1292 | [ω(C9H_2_)](19)+[ω(C11H_2_)](19)+[δ(C8H21O1)](13)+[δ(C12H20O6)](13)+  [γ(C9H_2_)](6)+ [γ(C11H_2_)](6) |
| 1307 | 1290 |  | 1282 | [ω(C11H_2_)](20)+[ω(C9H_2_)](19)+[δ(C12H20O6)](16)+[δ(C8H21O1)](16)+ [ν(CC)](10) |
| 1295 | 1278 | 1241 |  | [γ(C11H_2_)](21)+[γ(C9H_2_)](21)+[ν(C10O7)](12)+[δ(C8H21O1)](7)+[δ(C12H20O6)](7)+ [δ(C13H18O4)](6)+[δ(C10H19O7)](6) |
| 1233 | 1219 | 1216 | 1219 | [γ(C9H_2_)](35)+[γ(C11H_2_)](35)+[δ(C8H21O1)](7)+[δ(C12H20O6)](7) |
| 1192 | 1179 | 1169 | 1170 | [ν(CO)](35)+[δ(C13H18O4)](20)+[δ(C8H21O1)](6)+[δ(C12H20O6)](6) +[δ_sym_(C13O4)](5) |
| 1161 | 1149 |  |  | [ν(CO)](43)+[δ(C12H20O6)](7)+[δ(C8H21O1)](7)+[δ(C13H18O4)](7) |
| 1148 | 1137 | 1136 | 1145 | [ν(CO)](44)+[δ(C12H20O6)](11)+[δ(C8H21O1)](11)+[γ(C9H_2_)](7)+[γ(C11H_2_)](7) |
| 1127 | 1116 |  |  | [ν(CO)](43)+[δ(C13H18O4)](8)+[ν(CC)](16)+[δ(C10H19O7)](8) |
| 1075 | 1066 | 1080 | 1082 | [ω(C10C13O7)](27)+[ν(CC)](48)+[ω(C9H_2_)](5)+[ω(C11H_2_)](5)+[ω(C13O4)](5) |
| 1065 | 1056 | 1051 | 1052 | [ν(CC)](29)+[ρ(C10C13O7)]+10)+[ρ(C9H_2_)](8)+[ρ(C11H_2_)](8)+[ν(CO)](10)+ [δ(C10H19O7)](5) |
| 942 | 936 | 942 | 942 | [ρ(C11H_2_)](32)+[ρ(C9H_2_)](32)+[γ(C10C13O7)](7)+[ω(CO)](12) |
| 936 | 929 | 932 | 933 | [ν(C11C12)](50)+[δ_sci_(C10C9C11)](9)+[ν(CO)](14) |
| 910 | 904 | 904 | 904 | [ν(C10O7)](40)+[ρ(C11H_2_)](13)+[ρ(C9H_2_)](13)+[ω(CO)](12)+[ν(C10C13)](5) |
| 864 | 859 | 880 | 881 | [ν(CC)](48)+[δ_sci_(CCC)](12)+[ν(CO)](10) |
| 812 | 809 | 818 | 803 | [ω(CO)](39)+[ω(C10C13O7)](11)+[ν(C9C10)](7)+[ν(C10C11)](7)+δ_sci_(CCC)](12) |
| 754 | 752 | 781 | 783 | [ν(C10C13)](34)+[ρ(C13O4)](17)+[ν(C13O4)](9)+[δ(C13H18O4)](7) |
| 699 | 698 |  | 686 | [τ(CO)](40)+[ω(CO)](31) |
| 697 | 695 |  |  | [ω(CO)](39)+[τ(CO)](31)+[ρ(C11H_2_)](5)+[ρ(C9H_2_)](5) |
| 658 | 656 |  | 661 | [τ(CO)](61)+[ω(C13O4)](8)+δ_sym_(CO)](10) |
| 645 | 644 | 639 | 639 | [ρ(CO)](30)+[τ(CO)](24)+[δ_sym_(CO)](20) |
| 618 | 617 | 614 | 615 | [δ_sym_(C13O4)](22)+[δ_sci_(C10C13O7)](14)+[ρ(CO)](28)+[ν(C13O4)](5) |
| 578 | 578 | 598 | 597 | [τ(C13O4)](42)+[ρ(CO)](32) |
| 547 | 547 | 547 | 548 | [ω(CO)](28)+[τ(CO)](26)+[ρ(C13O4)](12)+[ρ(C10C13O7)](8) |
| 536 | 536 |  |  | [τ(CO)](33)+[ω(CO)](24)+[ρ(C11H_2_)](5)+[ρ(C9H_2_)](5) |
| 497 | 497 | 505 | 513 | [τ(C13O4)](19)+[ω(C13O4)](14)+[ρ(CO)](16)+[ω(C10C13O7)](8)+[ω(CO)](13)+ [γ(C10C13O7)](6) |
| 495 | 495 | 493 | 487 | [ρ(C13O4)](27)+[ρ(C10C13O7)](11)+[δ_sci_(C10C13O7)](10)+[ρ(C9H_2_)](7)+  [ρ(C11H_2_)](7)+[δ_sym_(C13O4)](7) |
| 457 | 458 | 428 | 420 | [τ(C10O7)](78) |
| 385 | 386 | - | 381 | [ρ(C10C13O7)](19)+[δ_sci_(C10C13O7)](11)+[δ_sym_(CO)](18)  [ρ(CO)](16)+[ρ(C9H_2_)](5)+[ρ(C11H_2_)](5)+[δ_sci_(C10C9C11)](5) |
| 363 | 364 | - | 365 | [δ_sym_(CO)](39)+[δ_sci_(C10C13O7)](15)+[ρ(C10C13O7)](13)+[ν(C10C13)](12)+  [ρ(C8O1)](6)+[ρ(C12O6)](6) |
| 348 | 349 | - | 349 | [γ(C10C13O7)](19)+[ω(C10C13O7)](16)+[τ(C10O7)](15)+[δ_sym_(CO)](18)+  [ρ(CO)](12) |
| 260 | 261 | - | 262 | [δ_sci_(C10C9C11)](18)+[δ_sym_(C13O4)](15)+[ρ(C10C13O7)](14)+[ρ(C13O4)](12)+ [ν(CC)](12) |
| 227 | 228 | - | 242 | [δ_sci_(C10C13O7)](23)+[δ_sci_(C10C9C11)](21)+[ρ(C10C13O7)](11)+[ρ(C13O4)](8)+ [δ_sym_(C13O4)](6) |
| 210 | 211 | - | 217 | [γ(C10C13O7)](42)+[ω(C13O4)](11)+[δ_sci_(CCC)](18)+[τ(C10O7)](6)+[δ_sym_(CO)]  (10) |
| 160 | 161 | - | 161 | [ω(C10C13O7)](22)+[δ_sci_(CCC)](44)+[γ(C10C13O7)](12)+[ω(C13O4)](5) |
| 128 | 129 | - | 127 | [τ(C10C13)](73)+[τ(C10O7)](11)+[δ_sci_(CCC)](10) |
| 105 | 106 | - | 108 | [δ_sci_(C10C9C11)](84) |
| 75 | 75 | - | - | [τ(CC)](78)+[τ(C10O7)](5) |
| 58 | 58 | - | - | [τ(CC)](79) |
| 36 | 36 | - | - | [τ(C8C9)](73)+[δ_sci_(CCC)](12) |
| 21 | 22 | - | - | [τ(C11C12)](82) |

**Table S4** Theoretical and experimental vibrational wavenumbers (cm^-1^) of monomer and dimer model of cocrystal and their assignments using B3LYP/6-311++G(d,p).

| **Scaled monomer** | **IR** | **Raman** | **Potential Energy**  **Distribution (**$\boldsymbol{\geq}\boldsymbol{5}\boldsymbol{\%}$**)** | **Scaled**  **dimer** | **Simplified**  **description of**  **modes** |
| --- | --- | --- | --- | --- | --- |
| 3559 | 3295 | - | [ν(O43H44)](100) | 3352,3108 | OH stretch |
| 3554 | 3168 | 3177 | [ν(O39H40)](100) | 3602, 3157 | OH stretch |
| 3513 | 3120 | 3121 | [ν(O33H34)](100) | 3511,3499 | OH stretch |
| 3124 | 3077 | 3079 | [ν(O26H27)](91)+[ν(N24H27)](5) | 3538,3078 | OH stretch + NH stretch |
| 3106 |  |  | R2[ν(C22H23)](93)+[ν(O26H27)](5) | 3097,3094 | Ring CH stretch + OH stretch |
| 3040 | 3042 | 3042 | R1[ν_a_(C2H_3_)](99) | 3038,3033 | Ring CH_3_ asym stretch |
| 3038 | 3025 | 3032 | R1[ν(C9H10)](86)+R1[ν(C9H12)](11) | 3032,2988 | Ring CH stretch |
| 3016 | 3015 | 3017 | R2[ν_a_(C18H_3_)](99) | 3017,3004 | Ring CH_3_ asym stretch |
| 3008 | 3008 | 3007 | R2[ν(C18H20)](52)+R2[ν(C18H21)](48) | 2997,2995 | Ring CH stretch |
| 2995 | 3000 | 2999 | R1[ν(C2H3)](50)+R1[ν(C2H5)](50) | 3001,2986 | Ring CH stretch |
| 2995 | 2992 | 2993 | [ν_a_(C35H_2_)](99) | 2994,2982 | CH_2_  asym stretch |
| 2987 | 2988 | 2982 | R1[ν(C9H12)](62)+R1[ν(C9H11)](35) | 2988,2978 | Ring CH stretch |
| 2976 | 2979 | 2973 | [ν_a_(C29H_2_)](98) | 3002,2948 | CH_2_  asym stretch |
| 2944 | 2959 | 2959 | [ν_s_(C35H_2_)](99) | 2942,2931 | CH_2_  sym stretch |
| 2938 | 2932 | 2932 | R1[ν_s_(C2H_3_)](77)+R2[ν_s_(C18H_3_)](22) | 2937,2931 | Ring CH_3_ sym stretch |
| 2938 |  |  | R2[ν_s_(C18H_3_)](78)+R1[ν(C2H5)](9)+R1[ν(C2H3)](9) | 2931,2928 | Ring CH_3_ sym stretch |
| 2924 | 2919 | 2920 | R1[ν_s_(C9H_3_)](100) | 2929,2920 | Ring CH_3_ sym stretch |
| 2913 | 2905 | 2909 | [ν_s_(C29H_2_)](99) | 2922,2906 | CH_2_  sym stretch |
| 1810 | 1738 | 1737 | [ν(C42=O45)](76)+[ρ(C42O43)](5) | 1784,1736 | C=O stretch |
| 1776 | 1697 | 1700 | [ν(C38=O41)](76)+[ρ(C38O39)](6)+[ν(C35C38)](5) | 1809,1689 | C=O stretch |
| 1759 |  |  | [ν(C25=O28)](73)+[ρ(C25O26)](6)+δ[C25H27O26](5) | 1752,1744 | C=O stretch |
| 1727 | 1642 | 1639 | R1[ν(C6=O7)](48)+R1[ν(C15=O16)](25)+R1[δ_tri_](9)+R1[ν(C14C15)]((5) | 1719,1646 | Ring C=O stretch |
| 1688 |  |  | R1[ν(C15=O16)]((40)+R1[ν(C6=O7)](28)+R1[ν(C14C15)](10)+R1[δ’_a_](7) | 1711,1681 | Ring C=O stretch |
| 1603 | 1606 | 1607 | [ν(C13=C14)](45)+R1[ν(C13N8)]((26)+R2[δ](7) | 1597,1592 | C=C stretch |
| 1549 | 1554 | 1556 | R2[ν(C14N17)](13)+R1[ν(C13N8)](11)+R2[ν(C13N24)](10)+R1[ν(C14C15)](8)+R2[δ_in_(C22H23)](8)+R1[δ_a_](7)+R1[δ_tri_](5)+R2[ν(C22N24)](5) | 1546,1545 | Ring CN stretch |
| 1510 | 1501 | 1502 | R2[ν(C22N17)](29)+R2[δ’_a_(C18H_3_)](11)+R2[δ_in_(C22H23)](9)+R2[δ_sym_(C18H_3_)](8)+R2[ρ(C18H_3_)](7)+R2[δ](7)+R2[ν(C22N24)](6) | 1508 | Ring CN stretch |
| 1491 |  |  | R1[δ_a_(C9H_3_)](56)+R2[δ’_a_(C18H_3_)](11)+R1[δ’_a_(C9H_3_)](9)+ R1[ρ(C9H_3_)](6) | 1484,1475 | Ring asym CH_3_ deformation |
| 1482 |  |  | R1[δ’_a_(C2H_3_)](51)+R1[δ_a_(C2H_3_)](17)+R1[ρ’(C2H_3_)](9)+R1[δ _sym_(C2H_3_)](8) | 1490,1479 | Ring asym CH_3_ deformation |
| 1480 | 1480 | 1482 | R1[δ’_a_(C9H_3_)](55)+R1[δ_a_(C9H_3_)](22)+R1[ρ’(C9H_3_)](6) | 1485,1465 | Ring asym CH_3_ deformation |
| 1471 | 1479 | 1477 | R2[δ’_a_(C18H_3_)](26)+R1[δ’_a_(C9H_3_)](11)+R2[δ_a_(C18H_3_)](8)+R1[δ’_a_(C2H_3_)](5)+R1[δ_sym_(C9H_3_)](5) | 1496,1463 | Ring asym CH_3_ deformation |
| 1470 | 1468 | 1465 | R1[δ_a_(C2H_3_)](69)+R1[δ’_a_(C2H_3_)](20)+R1[ρ(C2H_3_)](7) | 1473,1469 | Ring asym CH_3_ deformation |
| 1454 | 1456 | 1454 | R2[δ_a_(C18H_3_)](66)+R2[δ’_a_(C18H_3_)](22)+R2[ρ’(C18H_3_)](11) | 1469,1452 | Ring asym CH_3_ deformation |
| 1448 | 1445 | 1443 | δ_sci_[C35C38C32](78)+δ_sci_[C29C32C25](10)+δ_sci_[C35C38C32](3) | 1459,1450 | CCC scissoring |
| 1440 | 1438 | 1440 | R1[δ_sym_(C9H_3_)](26)+R1[δ_sym_(C2H_3_)](24)+R2[δ_sym_(C18H_3_)](12)+R2[ν(C22N24)](5) | 1443 | Ring sym CH_3_ deformation |
| 1432 |  | 1430 | R2[δ_sym_(C18H_3_)](61)+R1[δ_sym_(C9H_3_)](9)+[ν(C18N17)](7)+R1[δ_sym_(C2H_3_)](5)+R2[ν(C22N17)](5) | 1430,1427 | Ring sym CH_3_ deformation |
| 1427 |  |  | δ_sci_[C29C32C25](70)+δ_sci_[C35C38C32](10)+R1[δ_sym_(C2H_3_)](5) | 1431 | CCC scissoring |
| 1422 | 1421 | 1422 | R1[δ_sym_(C2H_3_)](38)+R1[δ_sym_(C9H_3_)](32)+δ_sci_[C29C32C25](7) | 1422,1418 | Ring sym CH_3_ deformation |
| 1397 | 1409 | 1410 | δ[C32H34O33](59)+ω[C29C32C25](12)+[ν(C32C35)](7)+ω[C32C29C35](5) | 1384 | CHO deformation |
| 1387 |  | 1387 | R2[ν(C14N17)](18)+R1[δ_sym_(C9H_3_)](10)+R2[ν(C22N24)](9)+R1[ν(C13N8)](7)+[ν(C18N17)](7)+R2[ν(C13N24)](6) | 1392,1380 | Ring CN stretch |
| 1383 | 1379 |  | ω[C35C38C32](27)+[ν(C35C38)](11)+[ν(C38O39)](7)+ω[C29C32C25](5)+δ[C38H40O39](5)+ϒ[C35C38C32](5) | 1376 | CCC wagging |
| 1366 |  | 1365 | R2[ν(C22N17)](18)+R2[ν(C22N24)](12)+R2[ν(C14N17)](11)+R2[ρ(C18H_3_)](6)+R2[δ’_a_(C18H_3_)](6)+R2[δ_in_(C18N17)](6)+R2[δ’](6)+R2[δ_in_(C22H23)](6) | 1361,1361 | Ring CN stretching |
| 1361 |  |  | δ[C25H27O26](23)+[ν(C25O26)](16)+ω[C29C32C25](15)+[ν(C25C29)](8)+[δ_sym_(C25O26)](5) | 1358,1353 | CHO deformation |
| 1343 | 1337 | 1347 | δ[C25H27O26](47)+ω[C29C32C25](16)+ϒ[C29C32C25](7)+δ[C32H34O33](6)+[ν(C25=O28)](5) | 1351,1348 | CHO deformation |
| 1330 | 1323 | 1325 | R2[ν(C22N24)](16)+R1[ν(C15N1)](11)+R2[ν(C13N24)](11)+[ν(C2N1)](10)+R1[ν(C6N8)](7)+R1[δ_in_(C6=O7)](6)+R1[δ_sym_(C2H_3_)](5) | 1323 | Ring CN stretch |
| 1322 | 1314 |  | δ[C42H44O43](19)+[ν(C32C42)](11)+δ[C38H40O39](10)+[ν(C42O43)](9)+[δ_sym_(C42O43)](7)+ϒ[C29C32C25](6)+[ν(C38O39)](5) | 1313 | CHO deformation |
| 1291 | 1292 | 1298 | ω[C35C38C32](43)+δ[C38H40O39](12)+ϒ[C35C38C32](8)+[ν(C32O33)](5) | 1298,1296 | CCC wagging |
| 1283 | 1285 | 1285 | R1[ν(C6N8)](18)+[ν(C9N8)](16)+R1[ν(C14C15)](11)+R2[ν(C13N24)](10)+R2[ν(C22N24)](6)+R1[ρ(C9H_3_)](5) | 1287,1283 | Ring CN stretch |
| 1281 | 1274 | 1277 | δ[C42H44O43](32)+ϒ[C35C38C32](13)+ϒ[C29C32C25](12)+δ[C38H40O39](11)+[ν(C32O33)](7) | 1280 | CHO deformation |
| 1254 | 1259 | 1258 | R1[ρ’(C2H_3_)](20)+R1[ν(C6N1)](16)+R1[ν(C15N1)](14)+R1[δ_in_(C2N1)](10)+R1[δ’_a_(C2H_3_)](6)+R1[ρ(C2H_3_)](6) | 1254,1249 | Ring CH_3_  rocking |
| 1246 | 1239 | 1239 | R2[δ_in_(C22H23)](20)+R1[ν(C6N1)](11)+R2[ν(C13N24)](10)+[ν(C2N1)](9)+R1[ρ(C9H_3_)](7)+R2[ν(C22N24)](6)+R2[δ’](5)+[ν(C18N17)](5) | 1245,1240 | Ring CH in plane deformation |
| 1224 | 1218 | 1232 | ϒ[C29C32C25](25)+ϒ[C35C38C32](21)+ω[C29C32C25](13)+[ν(C25O26)](12)+δ[C38H40O39](9) | 1224 | CCC twisting |
| 1212 | 1210 | 1207 | R1[δ_tri_](16)+[ν(C9N8)](15)+[ν(C18N17)](13)+R2[δ’](9)+[ν(C2N1)](8)+R1[ρ(C9H_3_)](5) | 1219,1210 | Ring trigonal deformation |
| 1199 | 1199 | 1195 | [ν(C25O26)](23)+ω[C29C32C25](7)+ϒ[C35C38C32](6)+R2[δ_in_(C22H23)](6)+δ[C38H40O39](5) | 1231 | CO stretch |
| 1194 | 1189 | 1182 | R2[δ_in_(C22H23)](18)+R2[ν(C13N24)](14)+R1[ρ(C9H_3_)](13)+[ν(C25O26)](11) | 1194,1192 | Ring CH in plane deformation |
| 1165 | 1174 | 1174 | ϒ[C29C32C25](18)+[ν(C32O33)](13)+[ν(C38O39)](12)+δ[C38H40O39](9)+[ν(C42O43)](5)+[ν(C25O26)](5) | 1175 | CCC twisting |
| 1142 |  |  | R1[ρ’(C9H_3_)](46)+R1[ρ(C2H_3_)](27)+R1[ρ’(C2H_3_)](9) | 1139 | Ring CH_3_ rocking |
| 1141 | 1133 | 1128 | [ν(C32O33)](16)+[ν(C29C32)](14)+[ν(C38O39)](13)+ϒ[C35C38C32](9)+[ν(C32C35)](6)+δ[C38H40O39](6)+δ_sci_[C32C29C35](5) | 1134,1128 | CO stretch |
| 1140 |  |  | R1[ρ’(C9H_3_)](38)+R1[ρ(C2H_3_)](35)+R1[ρ’(C2H_3_)](12) | 1134 | Ring CH_3_ rocking |
| 1136 |  |  | R2[ρ’(C18H_3_)](85)+R2[δ_a_(C18H_3_)](8) | 1140,1134 | Ring CH_3_  rocking |
| 1108 |  | 1110 | [ν(C42O43)](33)+δ[C42H44O43](19)+[ν(C32C42)](9)+[ν(C38O39)](7) | 1121 | CO stretch |
| 1083 | 1097 | 1086 | R2[ρ(C18H_3_)](54)+R2[ν(C22N17)](17)+R2[δ’](6) | 1100,1085 | Ring CH_3_ rocking |
| 1064 | 1064 | 1062 | R1[ρ(C9H_3_)](19)+R1[ν(C6N8)](19)+[ν(C2N1)](12)+R1[ν(C13N8)](8)+R2[ν(C14N17)](6)+R2[δ](5) | 1061 | Ring CH_3_ rocking |
| 1060 |  |  | [ν(C42O43)](15)+ρ[C29C32C25](11)+ρ[C32C29C35](9)+[ν(C29C32)](9)+ρ[C35C38C32](6)+[ν(C32O33)](6)+[ν(C38O39)](5) | 1072 | CO stretch |
| 1041 | 1043 | 1049 | [ν(C32C35)](35)+ω[C32C29C35](22)+[ν(C29C32)](15)+δ[C32H34O33](6)+[ω(C42O43)](5) | 1044 | CC stretch |
| 1032 | 1033 | 1031 | R1[ν(C15N1)](23)+R1[ν(C6N1)](23)+R1[ρ’(C2H_3_)](17)+[ν(C9N8)](7)+R1[ρ(C2H_3_)](6)+R2[δ’](5) | 1029,1028 | Ring CN stretch |
| 984 | 979 | 977 | [ν(C9N8)](25)+R2[δ](21)+[ν(C2N1)](15)+R1[ν(C6N8)](6)+R1[ν(C15N1)](6)+R1[δ’_a_](5) | 983,977 | CN stretch |
| 961 |  |  | [τ(C25O26)](80) | 933 | CO torsion |
| 941 | 940 | 941 | ρ[C35C38C32](35)+ρ[C29C32C25](18)+[τ(C25O26)](10)+ϒ[C32C29C35](5)+[ω(C25O26)](5) | 948,936 | CCC rocking |
| 930 | 930 | 932 | [ν(C2N1)](18)+R1[δin(C15=O16)](13)+R1[ν(C6N8)](9)+R1[ρ(C9H_3_)](9)+R2[ν(C14N17)](8)+R2[δ’](7)+R1[ν(C14C15)](5) | 931 | CN stretch |
| 925 |  | 928 | [ν(C25C29)](27)+[ν(C35C38)](19)+δ_sci_[C32C29C35](8)+[ν(C38O39)](7)+[ν(C25O26)](5)+δ_sci_[C35C38C32](5) | 926 | CC stretch |
| 905 | 911 | 908 | [ν(C32O33)](27)+ρ[C29C32C25](22)+[ω(C25O26)](7)+[ν(C29C32)](5)+[ν(C32C35)](5) | 905,903 | CO stretch |
| 867 | 886 | 887 | [ν(C35C38)](24)+[ν(C25C29)](22)+[ρ(C25O26)](10)+δ_sci_[C29C32C25](6)+[ν(C38O39)](6)+[ν(C29C32)](6)+δ_sci_[C35C38C32](5) | 884,860 | CC stretch |
| 830 | 827 | 829 | R2[oop(C22H23)](72)+R2[τ](13)+[τ(C22N24)](12) | 837 | Ring out of plane CH deformation |
| 816 | 815 |  | R1[δ_in_(C6=O7)](20)+R2[δ](20)+R1[δ_in_(C15=O16)](11)+R1[δ_in_(C2N1)](7)+[ν(C9N8)](6)+[ν(C18N17)](6)+R1[δ_tri_](6) | 814,809 | Ring in plane C=O deformation |
| 810 | 807 | 807 | [ω(C42O43)](25)+ω[C32C29C35](11)+[ω(C38O39)](10)+δ_sci_[C29C32C25](8)+[ν(C29C32)](8)+[δ_sym_(C25O26)](7)+δ_sci_[C35C38C32](5) | 812 | CO wagging |
| 761 | 764 | 766 | R1[oop(C15=O16)](38)+R2[τ’](25)+R1[puck](23)+R1[τ’_a_](6) | 760,756 | Ring out of plane C=O deformation |
| 751 | 745 | 743 | R1[oop(C6=O7)](15)+[ν(C18N17)](14)+R2[δ’](11)+R1[δ_in_(C6=O7)](9)+R1[δ_tri_](8)+R1[puck](6)+R1[δ_in_(C15=O16)](6)+R1[δ_in_(C9N8)](6) | 751,749 | Ring out of plane C=O deformation |
| 750 |  |  | R1[oop(C6=O7)](58)+R1[puck](17)+R1[oop(C2N1)](5) | 750 | Ring out of plane C=O deformation |
| 750 |  |  | [ν(C32C42)](29)+[ρ(C42O43)](13)+[ν(C42O43)](8)+[ω(C25O26)](7)+δ[C42H44O43](5) | 780,768 | CC stretch |
| 716 | 719 |  | [ω(C38O39)](22)+[ω(C42O43)](21)+[τ(C38O39)](18)+[τ(C42O43)](7) | 709,705 | CO wagging |
| 702 | 697 | 697 | R2[τ’](35)+R1[oop(C15=O16)](27)+R1[τ_a_](17)+[δ(C13=C14)](6)+R1[oop(C2N1)](5) | 702,701 | Ring torsion |
| 692 | 676 | 674 | [δ_sym_(C25O26)](17)+[ρ(C25O26)](17)+[ω(C25O26)](8)+δ_sci_[C29C32C25](6)+[τ(C38O39)](6) | 666 | CO sym deformation |
| 648 | 649 | 649 | [τ(C38O39)](38)+[ρ(C38O39)](10)+[δ_sym_(C38O39)](9)+[ρ(C25O26)](8)+[τ(C42O43)](8) | 657,654 | CO torsion |
| 643 | 638 | 639 | R1[δ_tri_](39)+[ν(C18N17)](13)+R2[δ’](10)+R2[ν(C13N24)](6)+R1[ν(C15N1)](5) | 648,643 | Ring tri deformation |
| 628 |  | 626 | [δ_sym_(C42O43)](15)+[ω(C25O26)](13)+[ρ(C42O43)](11)+δ_sci_[C32C29C35](10)+[τ(C38O39)](10)+[ρ(C25O26)](7) | 629 | CO sym deformation |
| 617 | 618 | 618 | R2[τ](78)+R2[oop(C18N17)](11) | 624,612 | Ring torsion |
| 588 | 594 | 592 | [τ(C42O43)](41)+[ρ(C38O39)](21)+[ρ(C25O26)](8)+[δ_sym_(C38O39)](7) | 595 | CO torsion |
| 565 | 573 | 585 | [ω(C25O26)](24)+[τ(C42O43)](10)+ρ[C29C32C25](8)+[τ(C38O39)](6)+[τ(C29C32)](5)+[δ_sym_(C25O26)](5)+[ω(C38O39)](5)+[τ(C25O26)](5) | 567 | CO wagging |
| 552 | 551 | 553 | R1[ν(C6N1)](11)+R1[ν(C15N1)](11)+[ν(C9N8)](11)+R1[ν(C14C15)](9)+R2[δ_in_(C18N17)](8)+R1[ν(C13N8)](7)+R2[ν(C14N17)](7)+R1[ν(C6N8)](6)+[ν(C2N1)](6) | 553,552 | Ring CN stretch |
| 537 | 538 |  | [τ(C32O33)](35)+[τ(C42O43)](12)+ρ[C29C32C25](7)+[ω(C38O39)](5)+δ_sci_[C32C29C35](5)+[τ(C38O39)](5) | 546 | CO torsion |
| 531 | 526 |  | [τ(C32O33)](26)+[ω(C25O26)](16)+ρ[C29C32C25](8)+[ω(C38O39)](7)+[τ(C29C32)](6)+[τ(C38O39)](5) | 526 | CO torsion |
| 502 | 500 | 515 | [ρ(C42O43)](20)+[τ(C32O33)](14)+ρ[C32C29C35](13)+[τ(C42O43)](6)+[ρ(C25O26)](5)+[ρ(C38O39)](5) | 511 | CO rocking |
| 486 | 488 | 488 | R1[δ_a_](9)+R1[δ’_a_](7)+[ρ(C42O43)](7)+ρ[C29C32C25](6)+[ω(C38O39)](5) | 488 | Ring asym deformation |
| 484 | 462 |  | R1[δ_a_](16)+R1[δ’_a_](11)+[ρ(C42O43)](5)+ρ[C29C32C25](5) | 486 | Ring asym deformation |
| 445 | 447 | 448 | R1[δ’_a_](35)+R1[δ_a_](15)+R1[ν(C6N1)](8)+R1[δ_in_(C6=O7)](8) | 446 | Ring asym deformation |
| 426 | 430 | 428 | R1[δ_in_(C6=O7)](36)+R1[δ_a_](15)+R2[δ_in_(C18N17)](8)+R2[δ](6)+R1[ν(C13N8)](6)+R1[δ’_a_](5) | 427,417 | Ring in plane C=O deformation |
| 406 | 408 |  | [δ_sym_(C38O39)](20)+[ρ(C38O39)](16)+ρ[C35C38C32](10)+ρ[C32C29C35](9)+[τ(C32C35)](8)+ϒ[C32C29C35](7)+δ_sci_[C32C29C35](6) | 387 | sym CO deformation |
| 397 | - | 394 | R1[δ_in_(C15=O16)](47)+R2[δ_in_(C18N17)](18)+R1[δ_a_](9)+R1[δ_in_(C2N1)](6) | 399,398 | Ring in plane C=O deformation |
| 369 | - | 375 | [δ(C13=C14)](26)+R1[oop(C9N8)](10)+[δ_sym_(C25O26)](8)+R2[oop(C18N17)](6)+R2[τ’](6)+R1[puck](6)+R1[τ_a_](5)+[δ(C25O26N24)](5) | 367 | C=C deformation |
| 362 | - | 362 | [δ(C13=C14)](20)+R1[oop(C9N8)](8)+R1[δ_in_(C2N1)](8)+[δ_sym_(C25O26)](7)+R2[τ’](5) | 363,358 | C=C deformation |
| 360 | - | 353 | R1[δ_in_(C2N1)](30)+[δ(C13=C14)](12)+R2[δ_in_(C18N17)](8)+R1[δ’_a_](5)+R1[oop(C9N8)](5) | 360,359 | Ring in plane CN deformation |
| 351 | - | 342 | ρ[C32C29C35](14)+δ_sci_[C32C29C35](11)+ϒ[C32C29C35](11)+[ν(C32C42)](7)+[δ_sym_(C25O26)](7)+ω[C32C29C35](6)+[τ(C29C32)](5) | 337 | CCC rocking |
| 309 | - | 303 | R1[δ_in_(C9N8)](62)+R1[δ_in_(C2N1)](14) | 307,305 | Ring in plane CN deformation |
| 273 | - | 283 | R1[oop(C2N1)](63)+R1[τ_a_](19)+R1[oop(C9N8)](6) | 281,271 | Ring out of plane CN deformation |
| 272 | - | 268 | ϒ[C32C29C35](16)+δ_sci_[C29C32C25](11)+R1[oop(C2N1)](9)+δ_sci_[C32C29C35](8)+[δ_sym_(C42O43)](8)+ρ[C32C29C35](5)+[ω(C42O43)](5) | 268 | CCC twisting |
| 261 | - | 254 | δ_sci_[C32C29C35](25)+δ_sci_[C29C32C25](16)+[δ_sym_(C42O43)](10)+[ν(C25C29)](8)+[δ_sym_(C25O26)](6)+[ν(C35C38)](6) | 241 | CCC scissoring |
| 226 | - | 238 | R1[oop(C9N8)](19)+R1[τ’_a_](17)+R2[oop(C18N17)](17)+δ_sci_[C32C29C35](7) | 228 | Ring out of plane CN deformation |
| 222 | - | 227 | R1[oop(C9N8)](11)+R2[oop(C18N17)](11)+ϒ[C32C29C35](10)+R1[τ’_a_](10)+δ_sci_[C32C29C35](9)+δ_sci_[C32C29C35](6)+δ_sci_[C35C38C32](6)+[δ_sym_(C38O39)](6) | 218 | Ring out of plane CN deformation |
| 210 | - | 211 | R2[δ_in_(C18N17)](36)+R1[δ_a_](9)+R2[δ](8)+R2[ν(C14N17)](7)+R1[δ’_a_](6)+R1[ν(C14C15)](5) | 213,210 | Ring in plane CN deformation |
| 194 | - | - | [τ(C29C32)](20)+ϒ[C32C29C35](12)+ω[C32C29C35](10)+[ν(N24H27)](9)+δ_sci_[C29C32C25](8)+[τ(C32C35)](5)+[τ(C25C29)](5) | 192 | CC torsion |
| 162 | - | - | R1[puck](47)+R1[oop(C2N1)](17)+[τ(C9N8)](9)+[δ(C13=C14)](5)+R1[oop(C9N8)](5) | 172,150 | Ring puckering |
| 144 | - | - | [τ(C9N8)](54)+R1[puck](11)+R1[oop(C9N8)](6) | 141 | CN torsion |
| 134 | - | - | [ν(N24H27)](30)+δ_sci_[C35C38C32](17)+R1[puck](11)+[τ(C9N8)](8)+R1[oop(C9N8)](5) | 148 | NH stretch |
| 125 | - | - | R1[oop(C9N8)](41)+R1[oop(C2N1)](13)+R1[τ_a_](13)+R2[oop(C18N17)](8)+[δ(C13=C14)](8)+R1[τ’_a_](7) | 134 | Ring out of plane CN deformation |
| 118 | - | - | R1[τ_a_](18)+[τ(C2N1)](17)+R1[oop(C9N8)](11)+[τ(C18N17)](7)+[δ(C13=C14)](6)+[τ(C25O26)](5)+[τ(C25O26)](5) | 126 | Ring asym torsion |
| 115 | - | - | [τ(C25O26)](30)+[τ(C25O26)](21)+[τ(C32C42)](12)+[τ(C29C32)](8)+[τ(C32O33)](8)+[ν(N24H27)](5) | 121 | CO torsion |
| 102 | - | - | [τ(C25O26)](37)+[τ(C25O26)](33)+[τ(C25C29)](8)+[δ(C22N24O26)](8)+[τ(N24O26)](5) | 117 | CO torsion |
| 91 | - | - | R1[τ_a_](31)+[τ(C2N1)](20)+R1[τ’_a_](15)+R1[puck](10)+[τ(C18N17)](5)+R1[oop(C2N1)](5) | 92 | Ring asym torsion |
| 87 | - | - | [ν(N24H27)](21)+δ_sci_[C32C29C35](12)+[τ(C25O26)](11)+[τ(C25O26)](10)+δ_sci_[C29C32C25](9)+δ_sci_[C35C38C32](7)+[τ(C29C32)](6)+[δ(C25O26N24)](5) | 91 | NH stretch |
| 84 | - | - | [τ(C29C32)](33)+[τ(C32C35)](21)+[τ(C32C42)](11)+[τ(C25C29)](5) | 86 | CC torsion |
| 83 | - | - | R1[puck](23)+[τ(C2N1)](21)+R1[oop(C2N1)](11)+R1[oop(C9N8)](7)+[τ(C18N17)](7)+R1[τ’_a_](6)+R1[τ_a_](5)+[δ(C13=C14)](5) | 79 | Ring puckering |
| 74 | - | - | [τ(C18N17)](40)+R2[oop(C18N17)](27)+R1[τ’_a_](22) | 73 | CN torsion |
| 57 | - | - | [τ(C32C35)](27)+[τ(C29C32)](15)+[δ(C25O26N24)](9)+[τ(C32C42)](9)+[τ(C25O26)](6)+[τ(C32O33)](5)+[δ(C22N24O26)](5) | 54 | CC torsion |
| 42 | - | - | [δ(C25O26N24)](17)+[τ(C29C32)](12)+[τ(C22N24)](11)+[τ(C32C42)](8)+δ[C25H27O26](7)+R1[τ_a_](7)+[τ(C32O33)](6) | 45 | CON deformation |
| 35 | - | - | [τ(C25C29)](24)+[τ(C35C38)](17)+[δ(C22N24O26)](13)+[τ(C25O26)](10)+[τ(C29C32)](7)+[τ(C32C42)](5)+[τ(N24O26)](4)+[τ(C32O33)](4) | 40,34 | CC torsion |
| 30 | - | - | [τ(C35C38)](40)+[τ(C32C42)](24)+[τ(C25C29)](11) | 28 | CC torsion |
| 18 | - | - | [τ(C25O26)](44)+[τ(C25O26)](16)+[τ(C25C29)](11)+[δ(C22N24O26)](9) | 20,17 | CO torsion |
| 13 | - | - | [τ(C22N24)](30)+[τ(N24O26)](21)+[δ(C25O26N24)](20)+[δ(C22N24O26)](11)+[τ(C25O26)](7) | 14 | CN torsion |
| 10 | - | - | [τ(N24O26)](31)+[τ(C25C29)](22)+[τ(C32O33)](9)+[τ(C35C38)](7) | 11,10 | NO torsion |

**Table S5.** Geometrical parameter (bond length) and topological parameters for bonds of interacting atoms of intra- and intermolecular hydrogen bonding of monomer model of cocrystal: electron density (ρ_BCP_), Laplacian of electron density (∇^2^ρ_BCP_), electron kinetic energy density (G_BCP_), electron potential energy density (V_BCP_), total electron energy density (H_BCP_) at bond critical point (BCP) and estimated interaction energy (E_int_).

| **Hydrogen bonds** | **Bond length (Å)** | **ρ_BCP_ (a.u.)** | **∇^2^ρ_BCP_ (a.u.)** | **G_BCP_ (a.u.)** | **V_BCP_ (a.u.)** | **H_BCP_ (a.u.)** | **E_int_ (kcal mol^−1^)** |
| --- | --- | --- | --- | --- | --- | --- | --- |
| (N24**···**H27) | 1.8038 | 0.0408 | 0.1007 | 0.0042 | −0.0336 | −0.0294 | −10.5422 |
| (O28**···**H34) | 2.0543 | 0.0223 | 0.0778 | −0.0014 | −0.0166 | −0.0180 | −5.2083 |
| (O26**···**H12) | 2.8862 | 0.0046 | 0.0176 | −0.0009 | −0.0027 | −0.0036 | −0.8785 |
| (O43**···**H11) | 2.9516 | 0.0035 | 0.0112 | −0.0005 | −0.0019 | −0.0024 | −0.6275 |

**Table S6.** Geometrical parameters for the existence of intra- and intermolecular hydrogen bonding interaction in monomer model of cocrystal: bond distance (Å), bond angle (°) and sum of van der Waals radii of interacting atoms (Å).

| **Hydrogen bonds (D−H···A)** | **d_D-H_ (Å)** | **d_H···A_ (Å)** | **d_D···A_ (Å)** | **D-H···A (°)** | **(r_H_ + r_A_) (Å)** |
| --- | --- | --- | --- | --- | --- |
| (O26−H27**···**N24) | 0.9919 | 1.8038 | 2.7871 | 170.6632 | 2.75 |
| (C9−H12**···**O26) | 1.0902 | 2.8862 | 3.6103 | 123.9699 | 2.72 |
| (C9−H11**···**O43) | 1.0923 | 2.9516 | 4.0113 | 163.5889 | 2.72 |
| (O33−H34**···**O28) | 0.9712 | 2.0543 | 2.7866 | 130.7056 | 2.72 |

**Table S7.** Geometrical parameters for intra- and intermolecular hydrogen bonding interaction in dimer model of cocrystal: bond distance (Å), bond angle (°) and sum of van der Waals radii of interacting atoms (Å).

| **Interactions (D−H∙∙∙A)** | **d_D−H_ (Å)** | **d_H∙∙∙A_ (Å)** | **d_D∙∙∙A_ (Å)** | **D−H∙∙∙A (°)** | **(r_H_ + r_A_) (Å)** |
| --- | --- | --- | --- | --- | --- |
| (C22-H23∙∙∙O43) | 1.0801 | 2.3402 | 3.2813 | 144.6513 | 2.72 |
| (C22-H23∙∙∙O90) | 1.0801 | 2.8593 | 3.7648 | 141.4640 | 2.72 |
| (O26-H27∙∙∙N24) | 0.9945 | 1.8125 | 2.8027 | 173.3576 | 2.75 |
| (C29-H30∙∙∙N24) | 1.0876 | 2.6221 | 3.5013 | 137.4372 | 2.75 |
| (O33-H34∙∙∙O28) | 0.9713 | 2.0982 | 2.8557 | 133.5626 | 2.72 |
| (O39-H40∙∙∙O52) | 0.9929 | 1.6916 | 2.6764 | 170.7294 | 2.72 |
| (C47-H50∙∙∙O41) | 1.0892 | 2.5521 | 3.5630 | 153.9591 | 2.72 |
| (O88-H89∙∙∙O41) | 0.9950 | 1.6898 | 2.6426 | 158.9258 | 2.72 |
| (O78-H79∙∙∙O90) | 0.9703 | 2.0515 | 2.6589 | 118.8209 | 2.72 |
| (C18-H19∙∙∙O90) | 1.0889 | 2.5247 | 3.5895 | 165.5398 | 2.72 |
| (O43-H44∙∙∙O86) | 0.9713 | 1.8132 | 2.7751 | 165.8548 | 2.72 |

**Table S8.** Geometrical parameter (bond length) and topological parameters for bonds of interacting atoms of intra- and intermolecular interactions of dimer model of cocrystal: electron density (ρ_BCP_), Laplacian of electron density (∇^2^ρ_BCP_), electron kinetic energy density (G_BCP_), electron potential energy density (V_BCP_), total electron energy density (H_BCP_) at bond critical point (BCP) and estimated interaction energy (E_int_).

| **Interactions** | **Bond length (Å)** | **ρ_BCP_ (a.u.)** | $\boldsymbol{\nabla}$**^2^ρ_BCP_ (a.u.)** | **G_BCP_ (a.u.)** | **V_BCP_ (a.u.)** | **H_BCP_ (a.u.)** | **E_int_ (kcal mol^−1^)** | **D−H∙∙∙A (°)** |
| --- | --- | --- | --- | --- | --- | --- | --- | --- |
| (C70=O73∙∙∙C87) | 2.8275 | 0.0118 | 0.0463 | −0.0017 | −0.0082 | −0.0099 | −2.5728 | 89.0108 |
| (C42-O43∙∙∙O90) | 3.1415 | 0.0059 | 0.0213 | −0.0006 | −0.0041 | −0.0047 | −1.2864 | 125.40 |

**Table S9** Second-order perturbation theory analyses of the Fock matrix, in the NBO basis for interactions in monomer model of cocrystal.

| **Donor NBO(i)** | | | **ED(i)/e** | | | | **Acceptor NBO(j)** | | **ED(j)/e** | | **E^(2)a^ (kcalmol^−1^)** | | **E(j)-E(i)^b^ (a.u.)** | | **F(i,j)^c^ (a.u.)** | |
| --- | --- | --- | --- | --- | --- | --- | --- | --- | --- | --- | --- | --- | --- | --- | --- | --- |
| **Within unit 1 (CAF)** | | | | | | | | | | | | | | | | |
| σ(C13=C14) | | 1.96627 | | | σ*(N17-C18) | | | | 0.01757 | | 5.54 | | 1.03 | | 0.068 | |
| π(C13=C14) | | 1.72849 | | | π*(C15=O16) | | | | 0.35221 | | 27.75 | | 0.30 | | 0.083 | |
| π(C13=C14) | | 1.72849 | | | π*(C22-N24) | | | | 0.46151 | | 13.11 | | 0.25 | | 0.054 | |
| π(C15=O16) | | 1.98208 | | | π*(C13=C14) | | | | 0.42146 | | 4.62 | | 0.38 | | 0.042 | |
| σ(C22-N24) | | 1.98122 | | | σ*(N8-C13) | | | | 0.03720 | | 7.08 | | 1.30 | | 0.086 | |
| π(C22-N24) | | 1.84327 | | | π*(C13=C14) | | | | 0.42146 | | 25.46 | | 0.34 | | 0.089 | |
| n(1)N1 | | 1.62540 | | | π*(C6=O7) | | | | 0.36988 | | 54.87 | | 0.27 | | 0.109 | |
| n(1)N1 | | 1.62540 | | | π*(C15=O16) | | | | 0.35221 | | 53.08 | | 0.27 | | 0.108 | |
| n(2)O7 | | 1.84020 | | | σ*(N1-C6) | | | | 0.08495 | | 26.28 | | 0.64 | | 0.118 | |
| n(2)O7 | | 1.84020 | | | σ*(C6-N8) | | | | 0.08486 | | 25.77 | | 0.65 | | 0.118 | |
| n(1)N8 | | 1.63568 | | | π*(C6=O7) | | | | 0.36988 | | 55.43 | | 0.27 | | 0.110 | |
| n(1)N8 | | 1.63568 | | | σ*(C9-H11) | | | | 0.01209 | | 5.06 | | 0.67 | | 0.057 | |
| n(1)N8 | | 1.63568 | | | π*(C13=C14) | | | | 0.42146 | | 49.50 | | 0.27 | | 0.106 | |
| n(2)O16 | | 1.85604 | | | σ*(N1-C15) | | | | 0.09625 | | 28.28 | | 0.65 | | 0.122 | |
| n(2)O16 | | 1.85604 | | | σ*(C14-C15) | | | | 0.05694 | | 17.30 | | 0.74 | | 0.103 | |
| n(1)N17 | | 1.52405 | | | π*(C13=C14) | | | | 0.42146 | | 29.65 | | 0.29 | | 0.084 | |
| n(1)N17 | | 1.52405 | | | π*(C22-N24) | | | | 0.46151 | | 65.68 | | 0.25 | | 0.114 | |
| n(1)N24 | | 1.88749 | | | σ*(C13=C14) | | | | 0.02655 | | 5.11 | | 0.97 | | 0.064 | |
| n(1)N24 | | 1.88749 | | | σ*(N17-C22) | | | | 0.02844 | | 5.84 | | 0.86 | | 0.065 | |
| π*(C22-N24) | | 0.46151 | | | π*(C13=C14) | | | | 0.42146 | | 47.05 | | 0.04 | | 0.060 | |
| **From unit 1 (CAF) to unit 2 (CA)** | | | | | | | | | | | | | | | | |
| σ (C9-H12) | 1.98866 | | | σ*(O26-H27) | | | | 0.05974 | | | | 0.10 | | 0.94 | | 0.009 |
| σ (C13-C14) | 1.96627 | | | σ*(O26-H27) | | | | 0.05974 | | | | 0.26 | | 1.15 | | 0.016 |
| σ (C13-N24) | 1.96627 | | | σ*(O26-H27) | | | | 0.05974 | | | | 0.31 | | 1.25 | | 0.018 |
| σ (N17-C22) | 1.98651 | | | σ*(O26-H27) | | | | 0.05974 | | | | 0.11 | | 1.28 | | 0.011 |
| σ(C22-N24) | 1.98122 | | | σ*(O26-H27) | | | | 0.05974 | | | | 0.30 | | 1.27 | | 0.018 |
| π(C22-N24) | 1.84327 | | | σ*(O26-H27) | | | | 0.05974 | | | | 0.13 | | 0.76 | | 0.009 |
| π (C22-N24) | 1.84327 | | | σ*(C29-H30) | | | | 0.01286 | | | | 0.08 | | 0.75 | | 0.007 |
| n(1)N24 | 1.88749 | | | σ*(O26-H27) | | | | 0.05974 | | | | 23.27 | | 0.83 | | 0.126 |
| n(1)N24 | 1.88749 | | | σ*(C29-H30) | | | | 0.01286 | | | | 0.11 | | 0.82 | | 0.009 |
| **Within unit 2 (CA)** | | | | | | | | | | | | | | | | |
| σ(O26-H27) | 1.98399 | | | | | σ*(C25=O28) | | | | 0.02596 | | 4.97 | | 1.37 | | 0.074 |
| σ(C29-H31) | 1.96117 | | | | | π*(C25=O28) | | | | 0.22028 | | 5.16 | | 0.54 | | 0.050 |
| σ(C35-H37) | 1.96271 | | | | | π*(C38=O41) | | | | 0.20034 | | 4.66 | | 0.55 | | 0.047 |
| n(1)O26 | 1.96947 | | | | | σ*(C25-C29) | | | | 0.06757 | | 6.15 | | 0.93 | | 0.068 |
| n(2)O26 | 1.79400 | | | | | π*(C25=O28) | | | | 0.2208 | | 46.14 | | 0.35 | | 0.114 |
| n(2)O28 | 1.85175 | | | | | σ*C25-O26) | | | | 0.08464 | | 29.85 | | 0.65 | | 0.126 |
| n(2)O28 | 1.85175 | | | | | σ*(C25-C29) | | | | 0.06757 | | 18.06 | | 0.62 | | 0.097 |
| n(2)O33 | 1.93962 | | | | | σ*(C29-C32) | | | | 0.03955 | | 6.98 | | 0.63 | | 0.060 |
| n(2)O33 | 1.93962 | | | | | σ*(C32-C42) | | | | 0.10016 | | 8.22 | | 0.63 | | 0.065 |
| n(1)O39 | 1.97822 | | | | | σ*(C38=O41) | | | | 0.02656 | | 6.88 | | 1.23 | | 0.082 |
| n(2)O39 | 1.81662 | | | | | π*(C38=O41) | | | | 0.20034 | | 41.94 | | 0.37 | | 0.111 |
| n(2)O41 | 1.84232 | | | | | σ*(C35-C38) | | | | 0.06097 | | 17.95 | | 0.64 | | 0.098 |
| n(2)O41 | 1.84232 | | | | | σ*(C38-C39) | | | | 0.09943 | | 33.31 | | 0.61 | | 0.130 |
| n(1)O43 | 1.97590 | | | | | σ*(C42=O45) | | | | 0.01994 | | 6.80 | | 1.28 | | 0.083 |
| n(2)O43 | 1.83765 | | | | | π*(C42=O45) | | | | 0.18340 | | 40.74 | | 0.36 | | 0.109 |
| n(2)O45 | 1.83266 | | | | | σ*(C32-C42) | | | | 0.10016 | | 20.96 | | 0.60 | | 0.102 |
| n(2)O45 | 1.83266 | | | | | σ*(C42-O43) | | | | 0.10777 | | 35.07 | | 0.59 | | 0.131 |

**Table S10** Second-order perturbation theory analysis of the Fock matrix, in the NBO basis for intra- and intermolecular interactions in dimer model of cocrystal.

| **Donor NBO (i)** | **ED(i)/e** | **Acceptor NBO (j)** | **ED(j)/e** | **E^(2)a^ (kcal mol^−1^)** | | **E(j)-E(i)^b^ (a.u.)** | **F(i,j)^c^ (a.u.)** |
| --- | --- | --- | --- | --- | --- | --- | --- |
| **Within unit 1 (CAF)** | | | | | | | |
| σ(C13=C14) | 1.96607 | σ*(N17-C18) | 0.01709 | 5.48 | 1.03 | | 0.068 |
| π(C13=C14) | 1.72807 | π*(C15=O16) | 0.35403 | 28.46 | 0.29 | | 0.083 |
| π(C13=C14) | 1.72807 | π*(C22-N24) | 0.45500 | 12.51 | 0.26 | | 0.053 |
| σC22-N24) | 1.98040 | σ*(N8-C13) | 0.03764 | 7.22 | 1.28 | | 0.086 |
| π(C22-N24) | 1.83438 | π*(C13=C14) | 0.42531 | 26.65 | 0.34 | | 0.090 |
| n(1)N1 | 1.62832 | π*(C6=O7) | 0.37656 | 55.74 | 0.27 | | 0.109 |
| n(1)N1 | 1.62832 | π*(C15=O16) | 0.35403 | 52.07 | 0.27 | | 0.107 |
| n(2)O7 | 1.84208 | σ*(N1-C6) | 0.08402 | 25.97 | 0.64 | | 0.118 |
| n(2)O7 | 1.84208 | σ*(C 6-N8) | 0.08390 | 25.47 | 0.65 | | 0.117 |
| n(1)N8 | 1.63675 | π*(C6=O7) | 0.37656 | 56.16 | 0.27 | | 0.111 |
| n(1)N8 | 1.63675 | σ*(C9-H12) | 0.01135 | 5.01 | 0.67 | | 0.057 |
| n(1)N8 | 1.63675 | π*(C13=C14) | 0.42531 | 48.42 | 0.28 | | 0.105 |
| n(2)O16 | 1.85568 | σ*(N1-C15) | 0.09747 | 28.61 | 0.64 | | 0.122 |
| n(2)O16 | 1.85568 | σ*(C14-C15) | 0.05631 | 17.11 | 0.74 | | 0.103 |
| n(1)N17 | 1.52068 | π*(C13=C14) | 0.42531 | 30.15 | 0.29 | | 0.084 |
| n(1)N17 | 1.52068 | π*(C22-N24) | 0.45500 | 66.72 | 0.25 | | 0.115 |
| n(1)N24 | 1.88713 | σ*(C13=C14) | 0.02668 | 5.19 | 0.96 | | 0.065 |
| n(1)N24 | 1.88713 | σ*(N17-C22) | 0.02807 | 5.75 | 0.87 | | 0.065 |
| **From unit 1 (CAF) to unit 2 (CA)** | | | | | | | |
| σ(C9-H11) | 1.98874 | σ*(O26-H27) | 0.06165 | 0.09 | 0.94 | | 0.008 |
| σ(C13=C14) | 1.96607 | σ*(O26-H27) | 0.06165 | 0.26 | 1.14 | | 0.016 |
| σ(C13-N24) | 1.97952 | σ*(O26-H27) | 0.06165 | 0.31 | 1.24 | | 0.018 |
| σ(N17-C22) | 1.98621 | σ*(O26-H27) | 0.06165 | 0.10 | 1.27 | | 0.010 |
| σ(C22-H23) | 1.98342 | σ*(C29-H30) | 0.01412 | 0.07 | 0.99 | | 0.008 |
| σ(C22-N24) | 1.98040 | σ*(O26-H27) | 0.06165 | 0.32 | 1.25 | | 0.018 |
| π(C22-N24) | 1.83438 | σ*(C29-H30) | 0.01412 | 0.39 | 0.75 | | 0.016 |
| n(1)N24 | 1.88713 | σ*(O26-H27) | 0.06165 | 23.64 | 0.82 | | 0.126 |
| n(1)N24 | 1.88713 | σ*(C29-H30) | 0.01412 | 0.16 | 0.83 | | 0.010 |
| **From unit 2 (CA) to unit 1 (CAF)** | | | | | | | |
| σ(O26-H27) | 1.98271 | σ*(C13-N24) | 0.01933 | 0.08 | 1.23 | | 0.009 |
| σ(C29-H30) | 1.97715 | σ*(C22-H23) | 0.02122 | 0.05 | 0.93 | | 0.006 |
| σ(C32-C42) | 1.96816 | σ*(C22-H23) | 0.02122 | 0.05 | 1.04 | | 0.006 |
| σ(O43-H44) | 1.98728 | σ*(C22-H23) | 0.02122 | 0.11 | 1.17 | | 0.010 |
| n(1)O26 | 1.96959 | σ*(C13-N24) | 0.01933 | 0.08 | 1.03 | | 0.008 |
| n(1)O43 | 1.96807 | σ*(N17-C22) | 0.02807 | 0.10 | 1.06 | | 0.009 |
| n(1)O43 | 1.96807 | σ*(C22-H23) | 0.02122 | 2.00 | 1.00 | | 0.040 |
| n(2)O43 | 1.81500 | σ*(C22-H23) | 0.02122 | 0.06 | 0.74 | | 0.006 |
| **Within unit 2 (CA)** | | | | | | | |
| n(2)O26 | 1.78513 | π*(C25=O28) | 0.20880 | 34.55 | 0.40 | | 0.105 |
| n(2)O28 | 1.85449 | σ*(C25-O26) | 0.08378 | 29.10 | 0.66 | | 0.125 |
| n(2)O28 | 1.85449 | σ*(C25-C29) | 0.06572 | 17.27 | 0.63 | | 0.095 |
| n(2)O33 | 1.94074 | σ*(C29-C32) | 0.03812 | 5.91 | 0.64 | | 0.055 |
| n(2)O33 | 1.94074 | σ*(C32-C42) | 0.10192 | 9.03 | 0.63 | | 0.068 |
| n(1)O39 | 1.96897 | σ*(C38=O41) | 0.02846 | 9.31 | 1.16 | | 0.093 |
| n(2)O39 | 1.75700 | π*(C38=O41) | 0.26222 | 59.85 | 0.32 | | 0.124 |
| n(2)O41 | 1.86752 | σ*(C35-C38) | 0.05407 | 12.16 | 0.68 | | 0.083 |
| n(2)O41 | 1.86752 | σ*(C38-O39) | 0.07263 | 26.82 | 0.72 | | 0.126 |
| n(1)O43 | 1.96807 | σ*(C42=O45) | 0.02129 | 8.03 | 1.24 | | 0.089 |
| n(2)O43 | 1.81500 | π*(C42=O45) | 0.20009 | 37.59 | 0.41 | | 0.112 |
| n(2)O45 | 1.84133 | σ*(C32-C42) | 0.10192 | 20.59 | 0.60 | | 0.101 |
| n(2)O45 | 1.84133 | σ*(C42-O43) | 0.09826 | 32.19 | 0.63 | | 0.129 |
| **From unit 2 (CA) to unit 3 (CAF)** | | | | | | | |
| π( C38=O41) | 1.99263 | σ*(C47-H50) | 0.01111 | 0.32 | 0.82 | | 0.014 |
| σ(O39-H40) | 1.98368 | σ*(C51=O52) | 0.08464 | 0.12 | 1.34 | | 0.012 |
| σ(O39-H40) | 1.98368 | π*(C51=O52) | 0.18665 | 0.17 | 1.40 | | 0.014 |
| n(1)O39 | 1.96897 | π*(C51=O52) | 0.18665 | 0.07 | 1.22 | | 0.009 |
| n(1)O41 | 1.95440 | σ*(C47-H50) | 0.01111 | 0.26 | 1.12 | | 0.015 |
| n(2)O41 | 1.86752 | σ*(C47-H50) | 0.01111 | 0.24 | 0.73 | | 0.012 |
| **From unit 2 (CA) to unit 4 (CA)** | | | | | | | |
| σ(C35-C38) | 1.97910 | σ*(O88-H89) | 0.05521 | 0.06 | 1.05 | | 0.007 |
| σ(C38-O39) | 1.99521 | σ*(O88-H89) | 0.05521 | 0.18 | 1.36 | | 0.014 |
| σ(C38=O41) | 1.99622 | σ*(O88-H89) | 0.05521 | 0.17 | 1.47 | | 0.014 |
| σ(O43-H44) | 1.98728 | σ*(C83=O86) | 0.02447 | 0.22 | 1.34 | | 0.015 |
| n(1)O41 | 1.95440 | σ*(O88-H89) | 0.05521 | 11.03 | 1.09 | | 0.098 |
| n(2)O41 | 1.86752 | σ*(O88-H89) | 0.05521 | 12.35 | 0.70 | | 0.085 |
| n(1)O43 | 1.96807 | σ*(C83=O86) | 0.02447 | 0.06 | 1.17 | | 0.008 |
| n(1)O43 | 1.96807 | σ*(O84-H85) | 0.01090 | 0.18 | 4.27 | | 0.025 |
| **From unit 3 (CAF) to unit 2 (CA)** | | | | | | | |
| σ(N46-C51) | 1.98551 | σ*(O39-H40) | 0.05150 | 0.06 | 1.25 | | 0.008 |
| σ (C47-H50) | 1.98671 | σ*(O39-H40) | 0.05150 | 0.05 | 0.92 | | 0.006 |
| σ (C51=O52) | 1.90227 | σ*(O39-H40) | 0.05150 | 0.40 | 1.21 | | 0.020 |
| π(C51=O52) | 1.81675 | σ*(O39-H40) | 0.05150 | 0.56 | 0.83 | | 0.020 |
| σ(C51-N53) | 1.98287 | σ*(O39-H40) | 0.05150 | 0.23 | 1.25 | | 0.015 |
| n(1)O52 | 1.95272 | σ*(O39-H40) | 0.05150 | 13.23 | 1.11 | | 0.108 |
| n(2)O52 | 1.85981 | σ*(O39-H40) | 0.05150 | 7.97 | 0.70 | | 0.068 |
| **Within unit 3 (CAF)** | | | | | | | |
| π(N46-C51) | 1.72918 | π*(N46-C51) | 0.32504 | 25.94 | 0.90 | | 0.138 |
| π(N46-C51) | 1.72918 | σ*(C51=O52) | 0.08464 | 20.04 | 0.85 | | 0.123 |
| π(N46-C51) | 1.72918 | π*(C51=O52) | 0.18665 | 63.49 | 0.92 | | 0.220 |
| π(N46-C51) | 1.72918 | π*(C60=O61) | 0.35546 | 43.39 | 0.27 | | 0.099 |
| σ(C51=O52) | 1.90227 | π*(N46-C51) | 0.32504 | 58.21 | 1.43 | | 0.274 |
| π(C51=O52) | 1.81675 | π*(N46-C51) | 0.32504 | 128.08 | 1.06 | | 0.341 |
| π(C51=O52) | 1.81675 | σ*(C51=O52) | 0.08464 | 55.67 | 1.01 | | 0.217 |
| π(C51=O52) | 1.81675 | π*(C51=O52) | 0.18665 | 18.75 | 1.07 | | 0.127 |
| π(C58=C59) | 1.72372 | π*(C60=O61) | 0.35546 | 28.99 | 0.29 | | 0.084 |
| π(C58=C59) | 1.72372 | π*(C67-N69) | 0.41953 | 13.21 | 0.27 | | 0.055 |
| σ(C67-N69) | 1.97874 | σ*(N53-C58) | 0.04134 | 7.92 | 1.28 | | 0.090 |
| π(C67-N69) | 1.82530 | π*(C58=C59) | 0.43317 | 27.64 | 0.33 | | 0.091 |
| n(2)O52 | 1.85981 | σ*(N46-C51) | 0.32504 | 20.35 | 0.69 | | 0.107 |
| n(2)O52 | 1.85981 | σ*(C51-N53) | 0.07015 | 23.27 | 0.70 | | 0.116 |
| n(1)N53 | 1.62315 | π*(N46-C51) | 0.32504 | 13.95 | 0.91 | | 0.102 |
| n(1)N53 | 1.62315 | π*(C51=O52) | 0.18665 | 5.09 | 0.93 | | 0.064 |
| n(1)N53 | 1.62315 | π*(C58=C59) | 0.43317 | 46.20 | 0.28 | | 0.103 |
| n(2)O61 | 1.85394 | σ*(N46-C60) | 0.09974 | 29.17 | 0.63 | | 0.123 |
| n(2)O61 | 1.85394 | σ*(C59-C60) | 0.05610 | 16.96 | 0.74 | | 0.103 |
| n(1)N62 | 1.53887 | π*(C58=C59) | 0.43317 | 30.43 | 0.29 | | 0.084 |
| n(1)N62 | 1.53887 | π*(C67-N69) | 0.41953 | 57.76 | 0.26 | | 0.111 |
| n(1)N69 | 1.92051 | σ*(N62-C67) | 0.03290 | 6.60 | 0.84 | | 0.067 |
| **From unit 3 (CAF) to unit 4 (CA)** | | | | | | | |
| n(1)O52 | 1.95272 | σ*(O84-H85) | 0.01090 | 0.08 | 4.38 | | 0.016 |
| **From unit 4 (CA) to unit 1 (CAF)** | | | | | | | |
| π(C87=O90) | 1.98987 | σ*(C22-H23) | 0.02122 | 0.12 | 0.81 | | 0.009 |
| n(1)O90 | 1.97521 | σ*(C18-H19) | 0.00612 | 0.72 | 1.12 | | 0.025 |
| n(1)O90 | 1.97521 | σ*(C22-H23) | 0.02122 | 0.36 | 1.12 | | 0.018 |
| n(2)O90 | 1.84581 | σ*(C18-H19) | 0.00612 | 0.20 | 0.69 | | 0.011 |
| **From unit 4 (CA) to unit 2 (CA)** | | | | | | | |
| σ(C77-C87) | 1.96794 | π*(C42=O45) | 0.20009 | 0.06 | 0.74 | | 0.006 |
| σ(C80-C83) | 1.97833 | σ*(O43-H44) | 0.03494 | 0.10 | 1.09 | | 0.009 |
| σ(C83-O84) | 1.99548 | σ*(O43-H44) | 0.03494 | 0.13 | 1.39 | | 0.012 |
| σ(C83=O86) | 1.99583 | σ*(O43-H44) | 0.03494 | 0.07 | 1.53 | | 0.009 |
| π(C83=O86) | 1.99354 | σ*(O43-H44) | 0.03494 | 0.30 | 0.83 | | 0.014 |
| σ (C87=O90) | 1.99546 | π*(C42=O45) | 0.20009 | 0.14 | 1.19 | | 0.012 |
| π(C87=O90) | 1.98987 | π*(C42=O45) | 0.20009 | 0.14 | 0.48 | | 0.008 |
| σ (O88-H89) | 1.98523 | σ*(C38=O41) | 0.02846 | 0.35 | 1.35 | | 0.019 |
| n(1)O86 | 1.96287 | σ*(O43-H44) | 0.03494 | 8.34 | 1.14 | | 0.087 |
| n(2)O86 | 1.85815 | σ*O43-H44) | 0.03494 | 3.42 | 0.72 | | 0.046 |
| n(2)O88 | 1.78192 | π*(C38=O41) | 0.26222 | 0.07 | 0.33 | | 0.004 |
| n(2)O90 | 1.84581 | π*(C42=O45) | 0.20009 | 0.16 | 0.37 | | 0.007 |
| **From unit 4 (CA) to unit 3 (CAF)** | | | | | | | |
| σ(C77-C87) | 1.96794 | σ*(N46-C47) | 0.01458 | 0.07 | 1.04 | | 0.008 |
| σ (C87=O90) | 1.99546 | σ*(N46-C47) | 0.01458 | 0.18 | 1.49 | | 0.015 |
| π(C87=O90) | 1.98987 | σ*(N46-C47) | 0.01458 | 0.10 | 0.79 | | 0.008 |
| n(2)O90 | 1.84581 | σ*(N46-C47) | 0.01458 | 0.10 | 0.68 | | 0.008 |
| **Within unit 4 (CA)** | | | | | | | |
| σ(C74-H75) | 1.96038 | π*(C70=O73) | 0.19125 | 5.19 | 0.55 | | 0.050 |
| σ(C80-H82) | 1.95868 | π*(C83=O86) | 0.22261 | 5.85 | 0.51 | | 0.051 |
| n(2)O71 | 1.82680 | π*(C70=O73) | 0.19125 | 39.83 | 0.36 | | 0.108 |
| n(2)O73 | 1.83449 | σ*(C70-O71) | 0.10004 | 33.75 | 0.60 | | 0.130 |
| n(2)O73 | 1.83449 | σ*(C70-C74) | 0.06789 | 18.92 | 0.62 | | 0.099 |
| n(2)O78 | 1.94633 | σ*(C74-C77) | 0.03701 | 7.27 | 0.65 | | 0.061 |
| n(2)O78 | 1.94633 | σ*(C77-C80) | 0.03458 | 5.77 | 0.65 | | 0.055 |
| n(1)O84 | 1.97714 | σ*(C83=O86) | 0.02447 | 7.10 | 1.23 | | 0.083 |
| n(2)O84 | 1.80188 | π*(C83=O86) | 0.22261 | 48.43 | 0.34 | | 0.116 |
| n(2)O86 | 1.85815 | σ*(C80-C83) | 0.05548 | 15.46 | 0.66 | | 0.092 |
| n(2)O86 | 1.85815 | σ*(C83-O84) | 0.08871 | 31.53 | 0.64 | | 0.129 |
| n(1)O88 | 1.96471 | σ*(C87=O90) | 0.08011 | 10.49 | 1.19 | | 0.100 |
| n(2)O88 | 1.78192 | π*(C87=O90) | 0.02738 | 54.51 | 0.34 | | 0.122 |
| n(2)O90 | 1.84581 | σ*(C77-C87) | 0.09241 | 19.27 | 0.61 | | 0.098 |
| n(2)O90 | 1.84581 | σ*(C87-O88) | 0.08011 | 26.40 | 0.69 | | 0.123 |

**Table S11.** Reactivity descriptors as Fukui functions ($f_{k}^{+}{, f}_{k}^{-}$), local softness ($s_{k}^{+}{, s}_{k}^{-})$, local electrophilicity indices ($\omega_{k}^{+}{, \omega}_{k}^{-})$ for CAF-CA monomer model of cocrystal using Hirshfeld atomic charges.

| **Atom no.** | $\mathbf{f}_{\mathbf{k}}^{\mathbf{+}}$ | $\mathbf{s}_{\mathbf{k}}^{\mathbf{+}}$ | $\boldsymbol{\omega}_{\mathbf{k}}^{\mathbf{+}}$ | **Atom no.** | $\mathbf{f}_{\mathbf{k}}^{\mathbf{-}}$ | $\mathbf{s}_{\mathbf{k}}^{\mathbf{-}}$ | $\boldsymbol{\omega}_{\mathbf{k}}^{\mathbf{-}}$ |
| --- | --- | --- | --- | --- | --- | --- | --- |
| O7 | 0.085277 | 0.015973 | 0.173813 | O7 | 0.04181 | 0.007831 | 0.085218 |
| N8 | 0.066893 | 0.01253 | 0.136343 | C13 | 0.038216 | 0.007158 | 0.077893 |
| C13 | 0.036122 | 0.006766 | 0.073625 | C15 | 0.064041 | 0.011995 | 0.13053 |
| C14 | 0.074966 | 0.014042 | 0.152797 | O16 | 0.074229 | 0.013904 | 0.151295 |
| O16 | 0.0724 | 0.013561 | 0.147567 | N17 | 0.035388 | 0.006628 | 0.072128 |
| C22 | 0.060469 | 0.011326 | 0.123249 | H19 | 0.036711 | 0.006876 | 0.074825 |
| H23 | 0.029933 | 0.005607 | 0.06101 | H20 | 0.035748 | 0.006696 | 0.072862 |
| O33 | 0.028556 | 0.005349 | 0.058203 | C22 | 0.109942 | 0.020593 | 0.224086 |
| O41 | 0.030774 | 0.005764 | 0.062724 | H23 | 0.073717 | 0.013808 | 0.150251 |
| O45 | 0.053165 | 0.009958 | 0.108362 | N24 | 0.031784 | 0.005953 | 0.064783 |

**Table S12.** Reactivity descriptors as Fukui functions ($f_{k}^{+}{, f}_{k}^{-}$), local softness ($s_{k}^{+}{, s}_{k}^{-})$, local electrophilicity indices ($\omega_{k}^{+}{, \omega}_{k}^{-})$ for dimer model of cocrystal using Hirshfeld atomic charges

.

| **Atom** | $\mathbf{f}_{\mathbf{k}}^{\mathbf{+}}$ | $\mathbf{s}_{\mathbf{k}}^{\mathbf{+}}$ | $\boldsymbol{\omega}_{\mathbf{k}}^{\mathbf{+}}$ | **Atom** | $\mathbf{f}_{\mathbf{k}}^{\mathbf{-}}$ | $\mathbf{s}_{\mathbf{k}}^{\mathbf{-}}$ | $\boldsymbol{\omega}_{\mathbf{k}}^{\mathbf{-}}$ |
| --- | --- | --- | --- | --- | --- | --- | --- |
| O90 | 0.0609 | 0.0114 | 0.1240 | H10 | 0.0781 | 0.0006 | 0.1591 |
| N53 | 0.0546 | 0.0102 | 0.1114 | N23 | 0.0543 | 0.0075 | 0.1107 |
| H89 | 0.0512 | 0.0096 | 0.1043 | O88 | 0.0401 | 0.0084 | 0.0818 |
| H64 | 0.0447 | 0.0084 | 0.0911 | H89 | 0.0372 | 0.0096 | 0.0759 |
| O88 | 0.0447 | 0.0084 | 0.0912 | H11 | 0.0359 | 0.0027 | 0.0732 |
| C54 | 0.0436 | 0.0082 | 0.0889 | H21 | 0.0347 | 0.0081 | 0.0706 |
| H21 | 0.0430 | 0.0081 | 0.0876 | H49 | 0.0335 | 0.0007 | 0.0683 |
| N24 | 0.0400 | 0.0075 | 0.0815 | H12 | 0.0326 | 0.0030 | 0.0664 |
| C83 | 0.0348 | 0.0065 | 0.0710 | C25 | 0.0285 | 0.0038 | 0.0580 |
| C63 | 0.0331 | 0.0062 | 0.0675 | O78 | 0.0246 | 0.0045 | 0.0501 |
